# Prediction of complications in health economic models of type 2 diabetes: a review of methods used

**DOI:** 10.1007/s00592-023-02045-8

**Published:** 2023-03-03

**Authors:** Xinyu Li, Fang Li, Junfeng Wang, Anoukh van Giessen, Talitha L. Feenstra

**Affiliations:** 1 Faculty of Science and Engineering, Groningen Research Institute of Pharmacy, University of Groningen, A. Deusinglaan1, 9713AV Groningen, The Netherlands; 2 Division of Pharmacoepidemiology and Clinical Pharmacology, Utrecht Institute for Pharmaceutical Sciences, Utrecht University, Utrecht, The Netherlands; 3 Expertise Center for Methodology and Information Services, National Institute for Public Health and the Environment, Bilthoven, The Netherlands; 4 Center for Nutrition, Prevention and Health Services Research, National Institute for Public Health and the Environment, Bilthoven, The Netherlands

**Keywords:** Decision model, Health economic model, Prediction model, Scoping review, Type 2 diabetes

## Abstract

**Aim:**

Diabetes health economic (HE) models play important roles in decision making. For most HE models of diabetes 2 diabetes (T2D), the core model concerns the prediction of complications. However, reviews of HE models pay little attention to the incorporation of prediction models. The objective of the current review is to investigate how prediction models have been incorporated into HE models of T2D and to identify challenges and possible solutions.

**Methods:**

PubMed, Web of Science, Embase, and Cochrane were searched from January 1, 1997, to November 15, 2022, to identify published HE models for T2D. All models that participated in The Mount Hood Diabetes Simulation Modeling Database or previous challenges were manually searched. Data extraction was performed by two independent authors. Characteristics of HE models, their underlying prediction models, and methods of incorporating prediction models were investigated.

**Results:**

The scoping review identified 34 HE models, including a continuous-time object-oriented model (*n* = 1), discrete-time state transition models (*n* = 18), and discrete-time discrete event simulation models (*n* = 15). Published prediction models were often applied to simulate complication risks, such as the UKPDS (*n* = 20), Framingham (*n* = 7), BRAVO (*n* = 2), NDR (*n* = 2), and RECODe (*n* = 2). Four methods were identified to combine interdependent prediction models for different complications, including random order evaluation (*n* = 12), simultaneous evaluation (*n* = 4), the ‘sunflower method’ (*n* = 3), and pre-defined order (*n* = 1). The remaining studies did not consider interdependency or reported unclearly.

**Conclusions:**

The methodology of integrating prediction models in HE models requires further attention, especially regarding how prediction models are selected, adjusted, and ordered.

**Supplementary Information:**

The online version contains supplementary material available at 10.1007/s00592-023-02045-8.

## Introduction

Global healthcare expenditure for diabetes showed a more than threefold increase from $232 billion in 2007 to $727 billion in 2017 for individuals aged 20–79 [1]. To help decision makers efficiently and explicitly allocate scarce resources across many interventions, health economic (HE) models, which evaluate the lifetime costs and benefits of interventions using a quantitative analysis framework, are widely used [2].

More than 90% of individuals with diabetes are diagnosed as type 2 diabetes (T2D) [3], and T2D affected nearly half a billion people worldwide in 2018 [4]. For T2D, several HE models exist and have been repeatedly applied in a wide range of settings to support decision making [5–7], such as the reimbursement of medications [8], prevention programs [9], and treatment strategies [10].

To simulate complications, HE models usually incorporate prediction models that mathematically combine multiple predictors to estimate the risk of diabetes-related events. For example, the UK Prospective Diabetes Study (UKPDS) risk engine [11] formulated mathematical models, with covariates such as diabetes duration, age, gender, body mass index, and glycated hemoglobin A1c (HbA1c) to estimate the probability of macrovascular and microvascular complications, such as myocardial infarction (MI), stroke, and ulcer. T2D affects multiple organ systems, resulting in numerous interdependent complications in nearly 20% of individuals [12, 13]. For example, the risk of atrial fibrillation is substantially higher following an MI [14], and a fourfold risk of stroke follows atrial fibrillation [15]. The common approach to considering this interdependency in simulations with HE models is to first properly estimate the prediction models one by one—for instance, stroke history is used as a covariate for MI—and then integrate the interdependent prediction models in the HE models, most often using random ordering of the prediction models to reduce bias [11]. Despite the simplicity of random ordering, this approach might ignore the causal relations of T2D pathology and result in inaccuracy [16], so it is important to evaluate alternative approaches for the integration of multiple prediction models for complications within HE models.

Several systematic reviews focusing on HE models or prediction models in T2D have been published, but none of them investigated the methodology of ordering prediction models. Those focused on the HE models mainly aimed to summarize [7], compare [17], and assess the available HE models [18–20]. Despite the availability of many prediction models [21–24], few have been applied in HE models. The most commonly used prediction models are the UKPDS [11, 25] and Framingham risk equations [26–28], but the selection criteria for prediction models remain unclear [29].

Therefore, the objective of this study is to assess how prediction models are incorporated into HE models for T2D and answer research questions regarding the selection and integration of prediction models in HE models. As a scoping review, we do not aim to identify and compare all HE models or prediction models for T2D or to declare one as the best. Instead, our goal is to understand how and why existing HE models incorporate prediction models as they do and to discuss challenges and possible solutions in the application of prediction models. This will inform existing and future HE models by providing insight into possible further improvements to incorporating prediction models.

## Methods

This study was conducted and reported following the Preferred Reporting Items for Systematic Reviews and Meta-Analyses extension for Scoping Reviews (PRISMA-ScR) [30–32] (Table S1) and registered with the Open Science Framework (https://osf.io/8bmjc).

### Literature search

A literature search was performed in PubMed, Web of Science, Embase, and Cochrane to identify published HE models for T2D since January 1, 1997 (the publication year of the model by Eastman et al. [33]). The last search was performed on November 15, 2022. The search strategy (Appendix S1) combined three elements indicating T2D, HE models and prediction models. In addition, the Diabetes Simulation Modeling Database [34] was screened to include its registered models, and all models participating in one or more past Mount Hood challenges were included based on challenge reports (Table S2).

### Inclusion and exclusion criteria

Studies were included if they described HE models that estimated future health outcomes for individuals with T2D by applying prediction models. Evidence-based transition probabilities were recognized as prediction models when there was at least one independent variable as a predictor, e.g., diabetes duration or HbA1c, otherwise the paper was excluded. Papers that re-applied existing HE models without adjustment were excluded. Additionally, papers concerning other types or stages of diabetes (e.g., type 1 diabetes or pre-diabetes), particular complications of diabetes (e.g., neuropathy), or a subgroup of individuals (e.g., overweight individuals) were excluded. Finally, papers were excluded if they were not in English or their full texts were not publicly accessible. The same screening criteria were used for title, abstract, and full text.

### Extracted information

A data extraction form including three key themes was constructed to collect and summarize information in a consistent and standardized format (Table S3). The three themes consist of:

1) Main HE models structure: Basic model structure, time horizon, cycle length, and taxonomy [35] based on (a) cohort- or individual-level, (b) continuous- or discrete-time, and (c) discrete event simulation or state transition model or otherwise, were summarized.

2) Complications and mortality: Health states or events, prediction models applied for each macrovascular and microvascular complication and mortality were extracted, including their characteristics, as well as the selection criteria used for the choice of prediction models, if any.

3) Methods of integrating prediction models: No taxonomy exists to categorize the methods used to integrate prediction models, so we considered the following key question when summarizing approaches: are the prediction models interdependent? If so, were prediction models run (a) simultaneously; (b) in a specific predetermined order or (c) in some other combination?

Additionally, we extracted information describing the various prediction models that were identified in the HE models, including their statistical model structure (e.g., Cox-regression or parametric regression), follow-up time, population, predictors, outcomes and methods for modeling treatment effects (Table S4).

Two reviewers (X.L. and F.L.) independently extracted and summarized information. Disagreements were resolved through discussion or consultation with a third reviewer (A.G.).

## Results

**Fig. 1:**
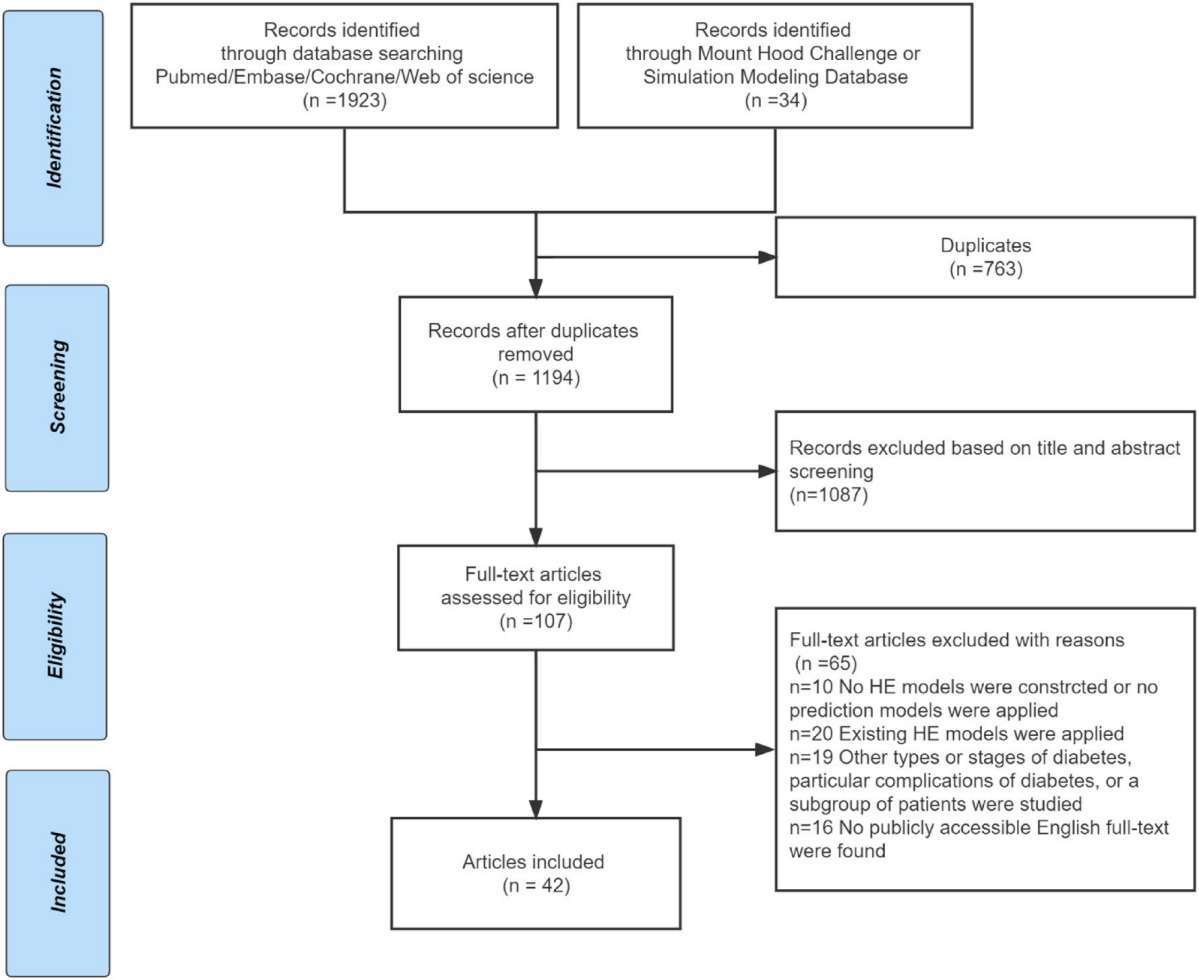
PRISMA flow chart for the literature review. Abbreviations: HE, Health economic

The selection process yielded 1923 citations from PubMed, Embase, Cochrane, and Web of science, and 34 citations from the Mount Hood Diabetes Simulation Modeling Database or challenges. After removing duplicates, screening based on title, abstract, and full text was performed and we identified 42 papers reporting on 34 key HE models (Fig. 1 and Table 1). Some models required more than one paper to understand and fully extract their information. All extracted information can be found in Table S3 and is summarized in Tables 2–4.

**Table 1:** Overview of models included in this study

Model (Abbreviation) with reference	Full name
*Health economic models*	
Archimedes [72, 73]	Archimedes model
BRAVO [74]	Building, relating, assessing, and validating outcomes diabetes microsimulation model
Cardiff [75]	The Cardiff diabetes model
Caro [76, 77]	NA: An economic evaluation model published by Caro et al
CDC [78–80]	NA: An economic evaluation model published by the CDC diabetes cost-effectiveness group
CHIME [81]	Chinese Hong Kong integrated modeling and evaluation
COMT [82]	Chinese outcomes model for type 2 diabetes
Cornerstone [83]	Cornerstone diabetes simulation model
DiDACT [84]	The diabetes decision analysis of cost—type 2 model
DMM [85]	The diabetes mellitus model
EAGLE [86]	Economic assessment of glycemic control and long-term effects of diabetes model
Eastman [33, 87]	NA: An economic evaluation model published by Eastman et al
ECHO [88]	The economic and health outcomes model of type 2 diabetes mellitus
GDM [16]	The global diabetes model
Grima [89]	NA: An economic evaluation model published by Grima et al
IHE [47, 90]	The Swedish institute for health economics diabetes cohort model
IMIB [91, 92]	NA: An economic evaluation model published by Palmer (Institute for Medical Informatics and Biostatistics) et al
IQVIA-CORE [93]	The IQVIA center for outcomes research diabetes model
JADE [5]	The Januvia diabetes economic model
JJCEM [94]	The Japan diabetes complications study/Japanese elderly diabetes intervention trial risk engine cost-effectiveness model
MICADO [95, 96]	Modelling integrated care for diabetes based on observational data
Michigan [97]	The Michigan model for diabetes
ODEM [98]	Ontario diabetes economic model
PRIME [53]	PRIME type 2 diabetes model
PROSIT [99]	The PROSIT disease modelling community (PROSIT in Latin means "it shall be useful")
RAMP-DM [100]	The risk assessment and management programme-diabetes mellitus
REDICT-DM [15]	Projection and evaluation of disease interventions, complications, and treatments–diabetes mellitus
Sheffield [101]	The Sheffield type 2 diabetes model
SPHR [102]	School for public health research diabetes model
Syreon [103]	The Syreon diabetes control model
Tilden [104]	NA: An economic evaluation model published by Tilden et al
TTM [105]	The Treatment Transitions Model
UKPDS-OM [25]	The UK Prospective Diabetes Study Outcomes Model
UKPDS-OM 2 [11]	The UK Prospective Diabetes Study Outcomes Model 2
*Prediction models*	
ADVANCE [106]	The model for cardiovascular risk prediction in action in diabetes and vascular disease: Preterax and Diamicron modified-release controlled evaluation
BRAVO (risk models) [74]	The prediction models of the building, relating, assessing, and validating outcomes diabetes microsimulation model based on the action to control cardiovascular risk in diabetes trial
CHIME (risk models) [81]	Risk prediction models used in the Chinese Hong Kong integrated modeling and evaluation model based on the Hong Kong clinical management system
EAGLE (risk models) [86]	Risk prediction models used in the economic assessment of glycemic control and long-term effects of diabetes model based on the Wisconsin epidemiological study of diabetic retinopathy, diabetes control and complications trial, and UK prospective diabetes study
Framingham [26–28]	Framingham risk models
Hong Kong registry risk models [107–109]	Risk prediction models based on Hong Kong registry data
JJRE [110]	Japanese elderly diabetes intervention trial risk engine
NDR (risk models) [111, 112]	Prediction models based on the Swedish national diabetes register
QRisk [113, 114]	Cardiovascular risk score based on the practices in England that had been using a clinical computer system developed by EMIS
RECODe [115]	Risk equations for complications of type 2 diabetes based on the action to control cardiovascular risk in diabetes trial
UKPDS (risk engine) [11, 25]	The UK prospective diabetes study risk engine

**Table 2:** General characteristics of 34 included type 2 diabetes health economic models (for more details see Tables S3 and S4)

		Count	Proportion (%)
Level of aggregation^1^	Individual-level	27	79
	Cohort-level	7	21
Prediction models applied^2^	UKPDS	20	59
	Framingham	7	21
	BRAVO	2	6
	NDR	2	6
	RECODe	2	6
	ADVANCE	1	3
	CHIME	1	3
	EAGLE	1	3
	Hong Kong registry risk models	1	3
	QRisk2	1	3
	JJRE	1	3
The method of integrating prediction models for macrovascular diseases^3^	Independent, Simultaneous evaluation	12	35
	Interdependent, Random order	12^4^	35
	Interdependent, Simultaneous evaluation (by a continuous model or lagged events)	4^5^	12
	Interdependent, 'Sunflower Method'	3^4^^, 5^	9
	Interdependent, Pre-defined order	1	3
	Interdependent, Unclear order	4	12

ADVANCE, model for cardiovascular risk prediction in Action in Diabetes and Vascular Disease: Preterax and Diamircon Modified-release Controlled Evaluation; BRAVO, the prediction models of Building, Relating, Assessing, and Validating Outcomes diabetes microsimulation model; CHIME, risk prediction models in Chinese Hong Kong Integrated Modeling and Evaluation; EAGLE, risk prediction models in Economic Assessment of Glycemic control and Long-term Effects of diabetes model; Framingham, Framingham risk models; JJRE, Japanese Elderly Diabetes Intervention Trial risk engine; NDR, prediction models from Swedish National Diabetes Register; QRisk2, Cardiovascular Risk Score 2; RECODe, Risk Equations for Complications of Type 2 Diabetes; UKPDS, The UK Prospective Diabetes Study risk engine

^1^The Global diabetes model (GDM) could be run either at the individual level or cohort level based on the user’s choice. Since its cohort-mode model was structured largely similar to the individual-level model, except that the cohort mode model applied mean values of risk factors for the cohort, the individual-level model of GDM was our study focus

^2^The number of HE models that applied this prediction model as part of their risk equations, does not add to 100%. Categories may overlap. Framingham, UKPDS and NDR contained several sets of risk equations. Details are listed in Table S3 and Table S4

^3^The method of integrating prediction models for microvascular disease (if different from macrovascular diseases) is listed in Table S3 to avoid confusion

^4^One model (Economic and Health Outcomes Model of Type 2 Diabetes Mellitus [ECHO-T2D]) can apply either 'sunflower method' or random order, by user’s choice

^5^One model (The Global Diabetes Model [GDM]) applied 'sunflower method' for initial events, and simultaneous evaluation (lagged events) for post-initial events

### Model classification and the use of prediction models within different model structures

**Fig. 2:**
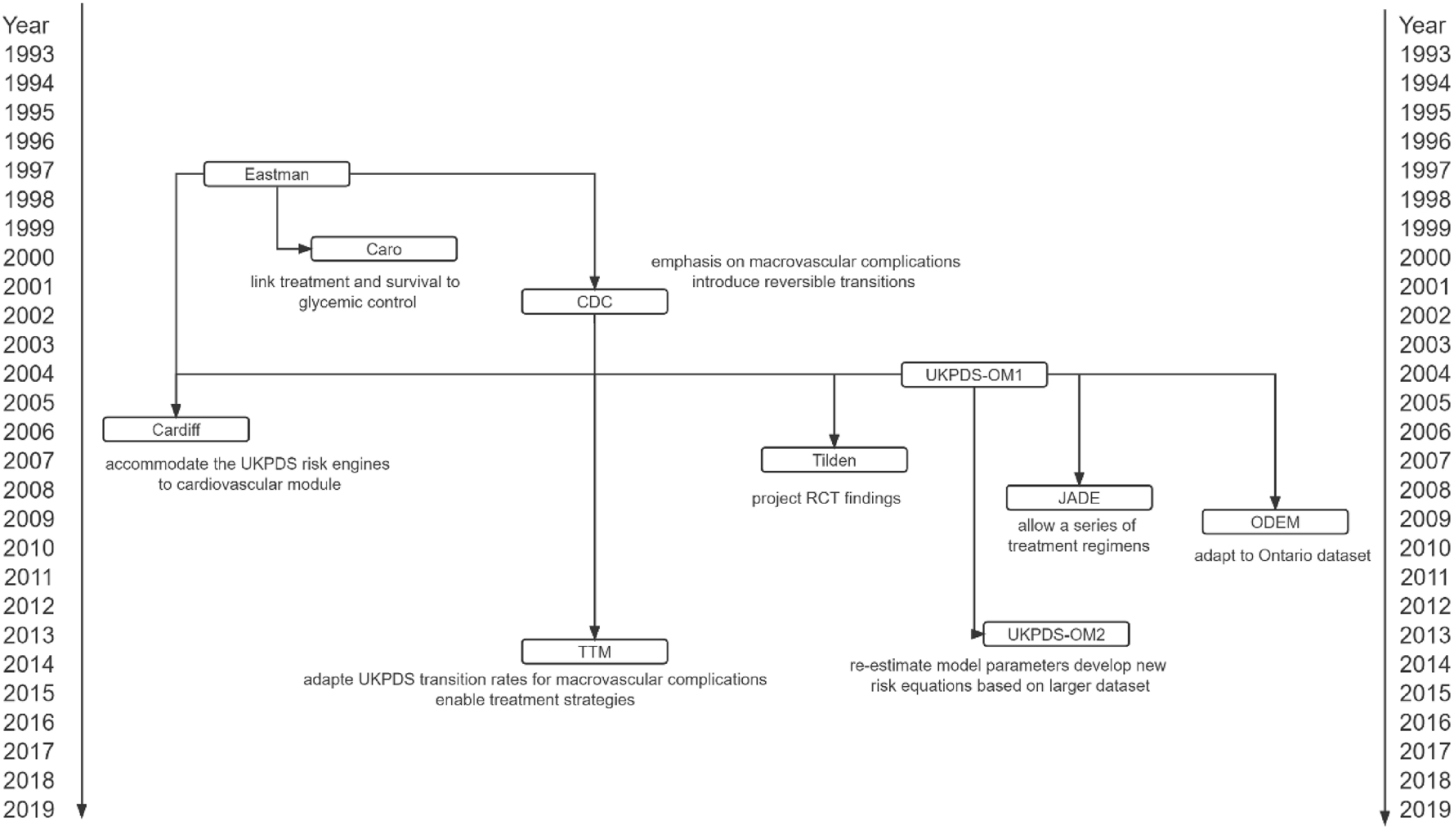
Development of some health economic models for type 2 diabetes. Abbreviations: Cardiff, The Cardiff Diabetes Model; Caro, an economic evaluation model published by Caro et al.; CDC, an economic evaluation model published by The CDC Diabetes Cost-effectiveness Group; Eastman, an economic evaluation model published by Eastman et al.; JADE, the Januvia Diabetes Economic model; ODEM, Ontario Diabetes Economic Model; RCT, Randomized Control Trials; Tilden, an economic evaluation model published by Tilden et al.; TTM, the Treatment Transitions Model; UKPDS-OM, The UK Prospective Diabetes Study Outcomes Model Connections were made based on the explicit reference indicating the extension in the subsequent paper. Otherwise, the model was not included in this graph

Developing HE models for diabetes is an iterative process, and many upgraded models have been built based on previous versions (Fig. 2). Many models consequently show a similar model structure (Tables 2 and 3).

**Table 3:** The model structure of 34 included health economic models

Model structure [35]	The number (proportion): Models	Description
*Individual-level*		
Continuous object-oriented Model (individual-level, continuous-time, object-oriented model)	*N* = 1 (3%): Archimedes[72]	Individual trajectories were simulated by physiology-based prediction equations (continuous-time models), simulating biological indicators and their interconnections at the level of organ systems
Discrete state transition model (individual-level, discrete-time, state transition model)	*N* = 11 (32%): Eastman[33, 87], Caro[76, 77], IQVIA-CORE[93], Michigan model[97], Tilden[104], ODEM[98], Sheffield[101], Syreon[103], PROSIT[99], and ECHO[88], and PREDICT[15]	Individual trajectories were simulated as changes from one discrete health state to another over a discrete and fixed time cycle, usually a year Transition probabilities were based on existing or self-developed prediction models, reflecting the risk to develop a certain complication during the model’s time cycle
Discrete event simulation model (individual-level, discrete-time, discrete event simulation model)	*N* = 15 (44%): GDM[16], UKPDS-OM1[25], EAGLE[86], Cardiff[75], JADE[5], DMM[85], UKPDS-OM2[11], TTM[105], SPHR[102], BRAVO[74], COMT[82], RAMP-DM[100], Cornerstone[83], CHIME[81], and PRIME[53]	Individual trajectories were simulated as a sequence of events by repeatedly applying prediction models, using a discrete time cycle, usually a fixed annual cycle Risks for events were based on existing or self-developed prediction models, reflecting the risk to develop a certain complication Main differences with the discrete state transition model: •Potentially infinite design space (No need for mutually exclusive health states [16].) •Flexibility in implementing changes and merging causal processes from trials [16]
*Cohort-level*		
Discrete state transition model (cohort-level, discrete-time, state transition model)	*N* = 7 (21%): IMIB[91, 92], DiDACT[84], CDC[78, 79], Grima[89], IHE[47, 90], MICADO[95, 96], and JJCEM[94]	Model the incidence, prevalence, and mortality of complications within assumed homogeneous cohorts, rather than simulating individual trajectories, which would account for heterogeneity Transition probabilities reflect the effect of covariates as follows: •Conditional relative risks (e.g., MICADO[95, 96]) •Stratification of probabilities by risk factor levels (e.g., IMIB[91, 92]) •Prediction models use the mean values of risk indicators for the cohort of interest (e.g., IHE[47, 90])

Archimedes, Archimedes model; BRAVO, Building, Relating, Assessing, and Validating Outcomes diabetes microsimulation model; Cardiff, The Cardiff Diabetes Model; Caro, an economic evaluation model published by Caro et al.; CDC, an economic evaluation model published by The CDC Diabetes Cost-effectiveness Group; CHIME, Chinese Hong Kong Integrated Modeling and Evaluation; COMT, Chinese Outcomes Model for Type 2 Diabetes; Cornerstone, Cornerstone Diabetes Simulation Model; DiDACT, The Diabetes Decision Analysis of Cost — Type 2 model; DMM, the Diabetes Mellitus Model; DMM, the Diabetes Mellitus Model; EAGLE, Economic Assessment of Glycemic control and Long-term Effects of diabetes model; Eastman, an economic evaluation model published by Eastman et al.; ECHO, The Economic and Health Outcomes Model of Type 2 Diabetes Mellitus; GDM, The Global Diabetes Model; Grima, an economic evaluation model published by Grima et al.; IHE, The Swedish Institute for Health Economics Diabetes Cohort Model; IMIB, an economic evaluation model published by Palmer (Institute for Medical Informatics and Biostatistics) et al.; IQVIA-CORE, The IQVIA CORE Diabetes Model; JADE, the Januvia Diabetes Economic model; JJCEM, The Japan Diabetes Complications Study/Japanese Elderly Diabetes Intervention Trial risk engine Cost-Effectiveness Model; MICADO, the Modelling Integrated Care for Diabetes based on Observational data; Michigan, The Michigan Model for Diabetes; ODEM, Ontario Diabetes Economic Model; PRIME, PRIME Type 2 Diabetes Model; PROSIT, the PROSIT Disease Modelling Community; RAMP-DM, the Risk Assessment and Management Programme-Diabetes Mellitus; REDICT-DM, PRojection and Evaluation of Disease Interventions, Complications, and Treatments–Diabetes Mellitus; Sheffield, the Sheffield type 2 diabetes model; SPHR, School for Public Health Research Diabetes Model; Syreon, the Syreon Diabetes Control Model; Tilden, an economic evaluation model published by Tilden et al.; TTM, the Treatment Transitions Model; UKPDS-OM, The UK Prospective Diabetes Study Outcomes Model; UKPDS-OM, The UK Prospective Diabetes Study Outcomes Model

Table 3 distinguishes four different model structures, including one continuous-time individual-level object-oriented model, 11 discrete-time individual-level state transition models, 15 discrete-time individual-level discrete event simulation models, and seven discrete-time cohort-level state transition models. The object-oriented model, Archimedes, applied differential equations as prediction models at the biological level. State transition models defined complications as states, with transition probabilities informed by prediction models, and thus the movement of an individual to another state indicates an event occurs in the current cycle. Discrete-time discrete event simulation models defined complications as events. Prediction models indicate the probability of event occurrence in a given time cycle. In the model simulation, these probabilities are compared with a random number drawn from a uniform distribution ranging from 0 to 1 to indicate whether an event occurs in the current cycle.

The most common states or events included in HE models were myocardial infarction (*n* = 23, 68%), heart failure (*n* = 21, 62%), and stroke (*n* = 14, 41%) for macrovascular complications, and retinopathy (*n* = 21, 62%), nephropathy (*n* = 19, 56%), and neuropathy (*n* = 18, 53%) for microvascular complications (Table S3).

### Application of prediction models

**Fig. 3:**
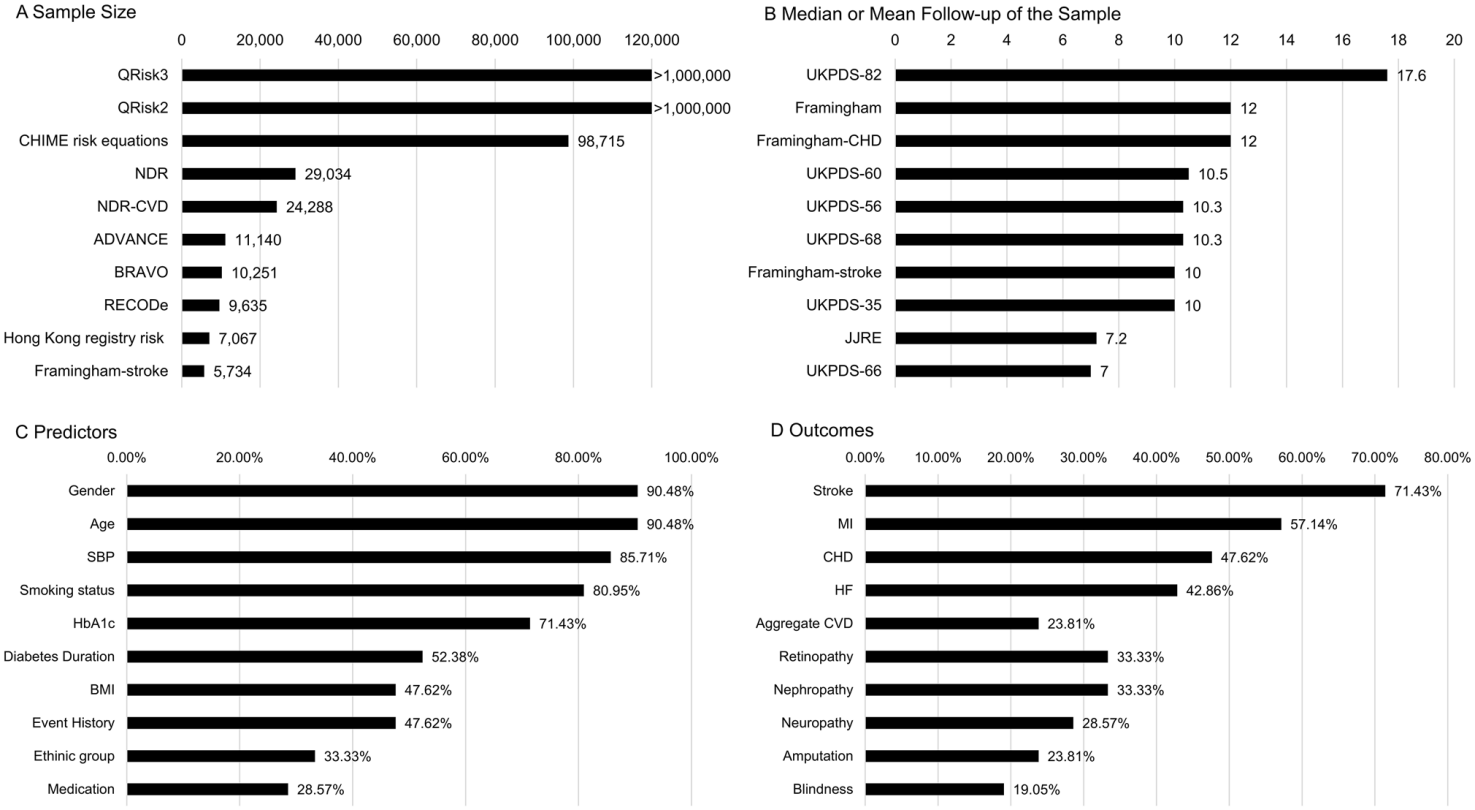
Characteristics of prediction models in type 2 diabetes health economic models. *Abbreviations:*ADVANCE, model for cardiovascular risk prediction in Action in Diabetes and Vascular Disease: Preterax and Diamicron Modified-release Controlled Evaluation; BMI, Body Mass Index; BRAVO, the prediction models of Building, Relating, Assessing, and Validating Outcomes diabetes microsimulation model; CHD, Congenital Heart Disease; CHIME, Chinese Hong Kong Integrated Modeling and Evaluation; CVD, Cardiovascular Disease; Framingham, Framingham risk models; HbA1c, Hemoglobin A1c; HF, Heart Failure; JJRE, Japanese Elderly Diabetes Intervention Trial risk engine; MI, Myocardial Infarction; NDR, prediction models from Swedish National Diabetes Register; QRisk, Cardiovascular Risk Score; RECODe, Risk Equations for Complications of Type 2 Diabetes; SBP, Systolic Blood Pressure; UKPDS, The UK Prospective Diabetes Study risk engine. Top 10 are listed for each characteristic, and full characteristics are listed in Tables S3 and S4

**Fig. 4:**
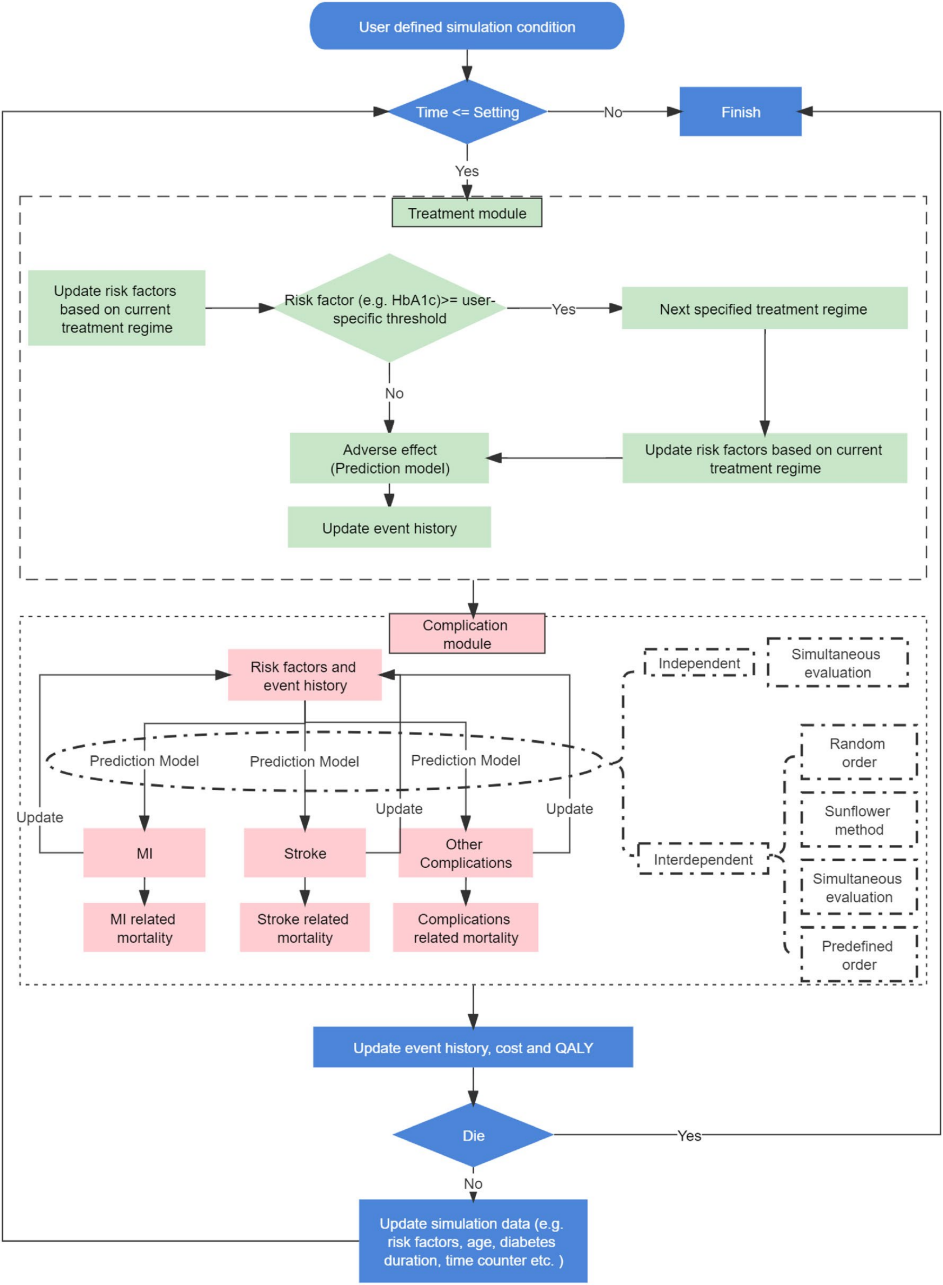
Flowchart of a general individual-level model structure. * Abbreviations:*HbA1c, Hemoglobin A1c; MI, Myocardial Infarction

Tables 4, 5 and 6, Fig. 3, and Table S4 provide an overview of prediction models that were employed in the HE decision models. Figure 4 indicates in general where and how the prediction models were applied in the HE models.

**Table 4:** Overview of frequently applied prediction models incorporated in type 2 diabetes health economic models

Study/Model (Publish year)	Basic model structure	The source of study population, main country, recruited/baseline period, median/mean follow-up time	HE models that incorporate this model. (number: names)
UKPDS risk engine [11, 25] (2004, 2013)	UKPDS OM1: Weibull proportional hazards regression UKPDS OM2: Weibull and Exponential proportional hazards regression (except ulcer applied logistic regression)	UKPDS, UK, UKPDS OM1: 1977–1991, 10.3 years UKPDS OM2: 1977–1991, 17.6 years	20: CDC, UKPDS-OM1, IQVIA-CORE, Michigan, EAGLE, Cardiff, Grima, Tilden, JADE, ODEM, Sheffield, DMM, UKPDS-OM2, TTM, IHE, SPHR, ECHO, Cornerstone, JJCEM, PRIME
Framingham [26–28] (1991, 1994, 1998)	Framingham: Non-proportional hazards Weibull accelerated failure time model Framingham-stroke and Framingham-CHD: Cox proportional hazards regression model	Framingham Heart Study and Framingham Offspring Study, US, Framingham: 1968–1975, 12 years Framingham-stroke: 1968–1975, 10 years Framingham-CHD: 1971–1974, 12 years	7: GDM, IMIB, Caro, DiDACT, CDC, IQVIA-CORE, SPHR
NDR risk models [111, 112] (2011, 2013)	NDR: Cox proportional hazards regression model NDR-CVD: Weibull proportional hazards model	NDR, Swedish, NDR: 2002–2003, 5 years NDR-CVD: 2003, 5 years	2: IHE, ECHO
RECODe [115] (2017)	Cox proportional hazards models	ACCORD, US, 2001–2005, 4.7 years^1^	2: COMT, PREDICT
BRAVO risk models [74] (2018)	Weibull proportional hazards model	ACCORD, US, 2001–2005, 3.7 years^1^	2: BRAVO, PRIME
EAGLE risk models [86] (2006)	Regression analyses (linear, exponential, and quadratic regression formulae) (details see Appendix S4)	WESDR, US, 1979, 1 year DCCT, US and Canada, 1983–1989, 6.5 years UKPDS, UK, 1977–1991,10 years	1: EAGLE
QRisk2 [113] (2007)	Cox proportional hazards regression model	All practices in England that had been using the EMIS computer system, UK, 1995–2007, 6.5 years	1: SPHR
Hong Kong registry risk models [107–109] (2007, 2008, 2008)	Cox proportional hazards regression model	Hong Kong Diabetes Registry, China, Stroke: 1995–2005, 5.4 years HF 1995–2005, 5.5 years CHD: 1995–2005, 5.4 years	1: PRIME
ADVANCE [106] (2011)	Cox proportional hazards regression model	ADVANCE, multiple countries in Asia, Australasia, Europe and Canada etc., 2001–2003, 4.5 years	1: ECHO
CHIME risk models [81] (2021)	Parametric proportional hazards models (exponential, log-logistic, log-normal, and Weibull)	Hong Kong Clinical Management System, China, 2006–2017, 4.1 years	1: CHIME
JJRE [110] (2013)	Cox proportional hazards regression model	JDCS, 1995–1996 and J-EDIT, 2001–2002, Japan, 7.2 years	1: JJCEM

ACCORD, Action to Control Cardiovascular Risk in Diabetes trial; ADVANCE, Action in Diabetes and Vascular Disease: Preterax and Diamircon Modified-release Controlled Evaluation; BRAVO, Building, Relating, Assessing, and Validating Outcomes diabetes microsimulation model; Cardiff, The Cardiff Diabetes Model; Caro, an economic evaluation model published by Caro et al.; CDC, an economic evaluation model published by The CDC Diabetes Cost-effectiveness Group; CHD, Congenital Heart Disease; CHIME, Chinese Hong Kong Integrated Modeling and Evaluation; COMT, Chinese Outcomes Model for Type 2 Diabetes; Cornerstone, Cornerstone Diabetes Simulation Model; CVD, Cardiovascular Disease; DCCT, Diabetes Control and Complications Trial; DiDACT, The Diabetes Decision Analysis of Cost — Type 2 model; DMM, the Diabetes Mellitus Model; DMM, the Diabetes Mellitus Model; EAGLE, Economic Assessment of Glycemic control and Long-term Effects of diabetes model; ECHO, The Economic and Health Outcomes Model of Type 2 Diabetes Mellitus; EMIS, a clinical computer system developed by EMIS; Framingham, Framingham risk models; GDM, The Global Diabetes Model; Grima, an economic evaluation model published by Grima et al.; HF, Heart Failure; IHE, The Swedish Institute for Health Economics Diabetes Cohort Model; IMIB, an economic evaluation model published by Palmer (Institute for Medical Informatics and Biostatistics) et al.; IQVIA-CORE, The IQVIA CORE Diabetes Model; JADE, the Januvia Diabetes Economic model; JDCS, Japan Diabetes Complications Study; J-EDIT, Japanese Elderly Diabetes Intervention Trial; JJCEM, The Japan Diabetes Complications Study/Japanese Elderly Diabetes Intervention Trial risk engine Cost-Effectiveness Model; Michigan, The Michigan Model for Diabetes; NDR, Swedish National Diabetes Register; ODEM, Ontario Diabetes Economic Model; PRIME, PRIME Type 2 Diabetes Model; QRisk2, Cardiovascular Risk Score 2; RECODe, Risk Equations for Complications of Type 2 Diabetes; REDICT-DM, PRojection and Evaluation of Disease Interventions, Complications, and Treatments–Diabetes Mellitus; Sheffield, the Sheffield type 2 diabetes model; SPHR, School for Public Health Research Diabetes Model; Tilden, an economic evaluation model published by Tilden et al.; TTM, the Treatment Transitions Model; UKPDS-OM, The UK Prospective Diabetes Study Outcomes Model; UKPDS, The UK Prospective Diabetes Study; UKPDS-OM, The UK Prospective Diabetes Study Outcomes Model; WESDR, Wisconsin Epidemiological Study of Diabetic Retinopathy

^1^ ACCORD participants were excluded due to missing candidate predictor variables in RECODe, causing the difference in median follow-up compared to BRAVO

**Table 5:** Overview of predictors used in prediction models incorporated in type 2 diabetes health economic models

	Gender	Age	SBP	Smoking status	HbA1c	Diabetes Duration	BMI	Race	Event History	Medication
Framingham [26]	Y	Y	Y	Y						
Framingham-stroke [27]	Y	Y	Y	Y					Y	Y
Framingham-CHD [28]	Y	Y	Y	Y						
UKPDS-35 [116]					Y					
UKPDS-56 [117]	Y	Y	Y	Y	Y	Y		Y		
UKPDS-60 [118]	Y	Y	Y	Y		Y				
UKPDS-66 – MI [119]		Y	Y		Y					
UKPDS-66 – Stroke [119]	Y		Y		Y				Y	
UKPDS-68 [25]	Y	Y	Y	Y	Y		Y	Y	Y	
UKPDS-82 [11]	Y	Y	Y	Y	Y	Y	Y	Y	Y	
NDR [112]	Y	Y	Y	Y	Y	Y	Y		Y	
NDR-CVD [111]	Y	Y	Y	Y	Y	Y	Y		Y	
EAGLE [86]	Y	Y	Y	Y	Y	Y		Y		
Hong Kong registry risk [107–109]	Y	Y		Y	Y	Y	Y		Y	
QRisk2 [113]	Y	Y	Y	Y			Y			Y
QRisk3 [114]	Y	Y	Y	Y			Y	Y		Y
ADVANCE [106]	Y	Y			Y	Y				Y
JJRE [110]	Y	Y	Y	Y	Y	Y	Y			
RECODe [115]	Y	Y	Y	Y	Y			Y	Y	Y
BRAVO [74]	Y	Y	Y	Y	Y	Y	Y	Y	Y	
CHIME risk equations [81]	Y	Y	Y	Y	Y	Y	Y		Y	Y

ADVANCE, model for cardiovascular risk prediction in Action in Diabetes and Vascular Disease: Preterax and Diamircon Modified-release Controlled Evaluation; BRAVO, the prediction models of Building, Relating, Assessing, and Validating Outcomes diabetes microsimulation model; CHD, Congenital Heart Disease; CHIME, risk prediction models in Chinese Hong Kong Integrated Modeling and Evaluation; EAGLE, risk prediction models in Economic Assessment of Glycemic control and Long-term Effects of diabetes model; Framingham, Framingham risk models; JJRE, Japanese Elderly Diabetes Intervention Trial risk engine; NDR, prediction models from Swedish National Diabetes Register; QRisk, Cardiovascular Risk Score; RECODe, Risk Equations for Complications of Type 2 Diabetes; UKPDS, The UK Prospective Diabetes Study risk engine

Top 10 most frequently applied predictors are listed here, and all predictors are listed in Table S4

**Table 6:** Overview of outcomes measured in prediction models incorporated in type 2 diabetes health economic models

	Stroke	MI	CHD	HF	CVD	Retinopathy	Nephropathy	Neuropathy	Amputation	Blindness
Framingham [26]	Y	Y	Y		Y					
Framingham-stroke [27]	Y									
Framingham-CHD [28]			Y							
UKPDS-35 [116]	Y	Y		Y		Y		Y	Y	
UKPDS-56 [117]		Y	Y							
UKPDS-60 [118]	Y									
UKPDS-66 – MI [119]		Y								
UKPDS-66 – Stroke [119]	Y									
UKPDS-68 [25]	Y	Y	Y	Y		Y	Y	Y	Y	Y
UKPDS-82 [11]	Y	Y	Y	Y		Y	Y	Y	Y	Y
NDR [112]	Y	Y	Y	Y						
NDR-CVD [111]					Y					
EAGLE [86]	Y	Y		Y		Y	Y	Y	Y	Y
Hong Kong registry risk [107–109]	Y		Y	Y						
QRisk2 [113]					Y					
QRisk3 [114]					Y					
ADVANCE [106]					Y					
JJRE [110]	Y		Y				Y			
RECODe [115]	Y	Y		Y		Y	Y			
BRAVO [74]	Y	Y		Y		Y	Y	Y		Y
CHIME risk equations [81]	Y	Y	Y	Y		Y	Y	Y	Y	

ADVANCE, model for cardiovascular risk prediction in Action in Diabetes and Vascular Disease: Preterax and Diamircon Modified-release Controlled Evaluation; BRAVO, the prediction models of Building, Relating, Assessing, and Validating Outcomes diabetes microsimulation model; CHD, Congenital Heart Disease; CHIME, risk prediction models in Chinese Hong Kong Integrated Modeling and Evaluation; CVD, Cardiovascular Disease; EAGLE, risk prediction models in Economic Assessment of Glycemic control and Long-term Effects of diabetes model; Framingham, Framingham risk models; HF, Heart Failure; JJRE, Japanese Elderly Diabetes Intervention Trial risk engine; MI, Myocardial Infarction; NDR, prediction models from Swedish National Diabetes Register; QRisk, Cardiovascular Risk Score; RECODe, Risk Equations for Complications of Type 2 Diabetes; UKPDS, The UK Prospective Diabetes Study risk engine

Top 10 most frequently estimated outcomes are listed here, and all outcomes are listed in Table S4

Older HE models estimated their own prediction models (e.g., Eastman, Archimedes, UKPDS and EAGLE), and newer HE models used or re-estimated existing prediction models, with a few exceptions (e.g., BRAVO and JJCEM). The UKPDS risk engine (*n* = 20, 59%) is the most frequently used set of prediction models (Table 2), followed by the Framingham, BRAVO, NDR, RECODe models. Updated versions of HE models tend to incorporate UKPDS risk engines rather than Framingham, which was developed 20 years ago, because UKPDS is more recent (published in 2013) and has a longer follow-up (1977–2007). The most recent prediction models are CHIME (cohorts observed from 2006 to 2017 and models published in 2021), RECODe, and BRAVO (both applied the Action to Control Cardiovascular Risk in Diabetes trial cohorts observed from 2001 to 2009 and published around 2018). The rationale for choosing particular existing prediction models is unclear in most HE models. Only the SPHR model explained that this was based on discussion with the stakeholder group regarding the suitability of settings, such as country, cohort characteristics, and covariate selection.

Many HE models applied different prediction models for macrovascular and microvascular disease risks (Table S3). Some existing prediction models (e.g., Framingham, ADVANCE and QRisk2) were only applicable for macrovascular disease. In many models, microvascular disease was estimated using diabetes duration stratified constant hazard ratios, assuming implicit exponential survival models. For example, Eastman calculated transition probabilities for various microvascular health states using published evidence-based diabetes duration-related hazard ratios (Algorithms see Appendix S2). Nine subsequent models (GDM, DiDACT, CDC, Cardiff, Sheffield, TTM, IHE, MICADO, and ECHO) also applied the Eastman transition rates or adjusted rates with the same algorithm, using newly published clinical evidence.

Most HE models split mortality into two components: cardiovascular disease (CVD) mortality and other mortality (Table S3). CVD mortality was informed by prediction models (e.g., Framingham, UKPDS, and RECODe), and other mortality was either derived from local mortality statistics (e.g., national life tables) or informed by mortality prediction models. Competing risk models were often applied to avoid overestimation of the mortality risk (e.g., DiDACT, CDC, and Michigan). As an exception, GDM applied one prediction model to estimate all-cause mortality, with a random number to define whether it was CVD or other mortality.

Two methods of re-calibrating prediction models to a specific setting were identified, either adjusting the default risk (e.g., EAGLE) or reconciling the transition probability in an iterative process until reproducing findings from an external population (e.g., Grima). Another adjustment option was providing users with choices of several prediction models (e.g., IQVIA-CORE, EAGLE, IHE, and ECHO). For example, ECHO enables the users’ choice between UKPDS, ADVANCE and NDR-CVD risk equations. Finally, rather than choosing between prediction models, PRIME implemented a model averaging approach based on the “distance” between the derivation cohort and the simulated cohort to evaluate individual-level risk informed by multiple risk models, including the UKPDS and BRAVO risk engines.

### Dealing with multiple complications and prediction models

Integrating selected and adjusted prediction models into a HE decision model is related to the HE model’s time cycle (Table S3). A fixed annual cycle was applied in the majority of models (n = 27, 79%), in which shorter cycles may be enabled for certain complications, such as a one-month cycle for neuropathy for COMT. Only DiDACT applied a 5-year cycle to simplify the model. A six-month cycle was applied in Tilden, JADE, and Syreon, and a one-month cycle in TTM and PREDICT. These shortened time cycles serve to ensure consistency with clinical trial data’s follow-up. Two methods were identified for incorporating prediction models with an original follow-up time other than the time cycle needed for the HE models. This was done either via algebraic compression and a constant risk assumption (e.g., GDM, RAMP, and PREDICT) or using the proportional hazard assumption and applying hazard ratios from the prediction models to survival over the duration of the time cycle from the HE model (e.g., SPHR), see Appendix S3 for algorithms.

Table 2 summarizes the methods of the combination of the prediction models. Many early HE models, especially those built before 2004, assumed no interdependence between different complications. GDM was the first model seen to assume interdependency of CVD events using the sunflower method. This method first predicted the occurrence of the next CVD event of any kind and used additional equations to predict which CVD event it would be, including combined events. This process was then repeated during the next cycle to estimate the order of events. For example, in SPHR, the QRisk2 equation estimated the CVD probability, and its nature (e.g., stroke or myocardial infarction, etc.) was determined separately by the published age- and gender-specific CVD distribution. ECHO also adopted this method as an optional choice for users.

An alternative approach is random order evaluation with interdependency. For example, IQVIA-CORE and UKPDS-OM tackled interactions among multiple complications by recording individuals’ event history in tracker variables and adjusting the risk of other complications accordingly, using dummies reflecting pre-existing complications in prediction models. To avoid possible bias, during simulations, the order of prediction models is randomly assigned for each time cycle.

A third approach is the simultaneous evaluation by lagged events information to inform on interdependencies. This approach avoids considering the interdependency of events occurring in the current cycle (e.g., MICADO). A predefined order, as the fourth approach, was applied in only JJCEM, in which the prediction model of amputation, which included retinopathy as a predictor, was run as the final model.

#### Treatment effects

Prediction models might be estimated from data with different treatments in place. Treatment effects were either included explicitly as a dummy variable, or implicitly as impact on risk factors. Most prediction models identified in health economic HE models only used dummy variables to reflect the use of antihypertensive medication (Framingham-stroke, ADVANCE, and QRisk2), while the effect of glucose control treatment was consistently modeled implicitly via impact on risk factors (e.g., UKPDS and JJRE). Two exceptions are that CHIME risk equations and RECODe include both antihypertensives and oral diabetes drug as dummy variables (Table S4). Statin use was also included in only these two risk equations (CHIME and RECODe), though ADVANCE included statin in the variable selection phase but it was finally dropped by stepwise approach.

Two methods were identified for modeling treatment effects while applying prediction models that do not explicitly consider treatment effects as dummy variables. One method applies relative risks or conditional probabilities (e.g., treatment-specific states and transition probabilities applied in IMIB). The other method estimates the effect of treatment on underlying risk factors (e.g., in Cardiff, the effect of medication on HbA1c was modeled via an update of HbA1c levels in a treatment module that, in turn, altered the probabilities of events, see Fig. 4). Treatment modules may also allow treatment switches, mimicking clinical practice. The trigger for this switch depended on an evidence-based transition matrix (e.g., DiDACT) or on individuals’ clinical indicators, such as a specific HbA1c level or diabetes duration (e.g., JADE).

Treatment compliance was considered in 6 (18%) models (Eastman, GDM, Michigan model, Tilden, Syreon, ECHO), by specifying the rate of individual compliance or simulating HbA1c levels between standard care (e.g., 10%) and comprehensive care (e.g., 7.2%).

## Discussion

We found four solutions for dealing with the interdependency of prediction models in HE simulation models. All approaches required several assumptions, and no new approaches were introduced in recent years. For many models, it was difficult to determine the exact methods applied because of the lack of transparency in reporting and the ambiguity of the terminology applied.

The pros and cons of various HE modeling structures have been widely discussed [7, 36, 37]. Our study investigated HE models from the perspective of incorporating prediction models. Individual-level discrete event simulation models would be the most straightforward structure, because individual-level models can be well informed directly by common prediction models, while cohort-level models require extra implementation steps (e.g., converting information from prediction models into relative risks or using mean risk factor values to inform prediction models). Additionally, as a result of Markov property assumptions, state transition models usually cannot easily accommodate prediction models that explicitly include duration, while discrete event simulation models can easily keep track of time as an attribute and hence directly use time-to-event prediction models.

Confirming previous studies [38, 39], we did not find a clear preference for certain prediction models. The likely explanation of UKPDS risk engine being the most commonly used prediction model, is that it is the first risk engine developed in a T2D population, covers most T2D complications, and has a high degree of transparency in describing its algorithm and coefficients [25]. However, the rationale for adopting specific prediction models in HE models has been underreported.

Being referenced or recommended in clinical guidelines could be one rationale for incorporating specific prediction models into HE models [40]. For instance, a risk calculator [41] is recommended for estimating the risk of ASCVD in American diabetes guidelines [42] and the UKPDS risk engine is referenced to measure CVD risk in European diabetes guidelines [43]. Since clinical prediction models have higher calibration requirements than HE models [44], such inclusion in guidelines could be seen as support for these prediction models. However, clinical prediction models and HE models may deviate in requirements regarding variable selection. Clinical prediction models often prefer including fewer predictors, which are available in routine care, whereas HE models may want to cover all of their modeled risk factors. When using clinical trials as main source of input this might be a wider range of predictors than those in routine care. In addition, for clinical application, discrimination next to calibration is very important [45], while for HE models, with their focus on aggregate outcomes, calibration-in-the-large will be the most important [46]. Due to these distinct scopes and requirements, clinical guideline-recommended prediction models are not always the best fit for HE models. For example, NDR performed better than UKPDS risk model for well-treated individuals, whereas UKPDS risk model performed better for the older UK cohort, indicating that the choice should reflect the specifics of the application [47].

In our opinion, several criteria could help select suitable prediction models as follows (multiple sets of prediction models can be chosen at the same time):

1. Time period: The UKPDS risk engine allows to validate modeling a population for a long-time horizon (17.6-year median follow-up), but covers a somewhat older time period (1977–2007). CHIME risk model suits well with short-term and recent time horizons (4-year mean follow-up until 2017).
2. Population: JJRE, CHIME, and the Chinese Hong Kong registry risk models suit well with an Asian population; NDR suits well with a European population; QRisk and UKPDS were developed in UK populations; BRAVO and RECODe were developed in US populations. If a mixture of multiple ethnic groups is of interest, UKPDS, EAGLE, QRisk, RECODe, and BRAVO which consider ethnic groups as a predictor, are suitable.
3. The available predictors are listed in Table 5 and could guide choice of prediction model(s). Of note, unavailable risk factors, such as white blood cell counts, may be imputed, enlarging the applicability of prediction models [48]. If information about both events and medication use is available, CHIME and RECODe are suitable.
4. Outcomes of interest can be found in Table 6. NDR, ADVANCE, and QRisk predict the aggregate CVD, while UKPDS, BRAVO, CHIME, and RECODe predict each separate CVD event (i.e., MI, stroke and others). For prediction of subsequent events (i.e., the second or next time of occurring), NDR and UKPDS offer most details. If microvascular diseases are of interest, UKPDS, EAGLE, BRAVO, and CHIME suit well.

Once prediction models are selected, properly incorporating them into HE models requires attention to recalibration and adjustment. When data are available, recalibrating prediction models is important if the cohort of deriving prediction models differs from that of the application at hand in a HE model. For example, UKPDS-OM2 poorly predicted the CANVAS program outcomes, but recalibrating intercepts and refitting the coefficients, while preserving the UKPDS-OM2 structure, substantially improved the fit [49]. That is, the recalibration of the prediction models based on available data or characteristics to the setting of interest involves adjusting the intercept (for logistic regression models) or the baseline survival function (for survival regression models) and adjusting regression coefficients for prediction models [24, 50–52]. Furthermore, especially when data for recalibration are unavailable, applying different sets of prediction models in HE models for the same outcome might help to overcome differences between populations. The weighted model averaging approach [53] could be applied to summarize multiple predictions. Alternatively, different prediction models could inform best-case or worst-case risk predictions and enable quantifying the structural uncertainty caused by prediction model choice [54–56]. This would inform HE model users better than a single prediction model.

Interdependency is increasingly incorporated when combining prediction models in HE models, but the order problem currently shows only four solutions: random ordering, the sunflower method, using lagged events or using a predefined order. Random ordering is the most common approach for recent HE models in T2D. Despite its advantage of simplicity, it might ignore potential biologically more plausible sequences of T2D complications. Alternative approaches therefore deserve further investigation, and we recommended to use them in different study designs accordingly and compare results to random ordering, to check which works best:

1. When the HE model is defined in continuous time: Use the vertical modeling approach [57, 58], as the continuous-time version of the sunflower method in the statistical analysis to derive prediction models. Both methods decompose the joint probability by looking first at the time of the event and then its cause at the time of failure based on observable quantities, such as relative cause-specific hazards [16, 57, 58]. However, vertical modeling is a continuous-time model that integrates time-proportional hazards and logistic regression with covariates [57, 58]. The sunflower method is a discrete-time method that compares the estimated time-related incidence rate to the relative event frequency [16].
2. When the HE model is defined in discrete time, and the event progression is moderate during one cycle: Use linked-equations with a time-lagging structure to minimize the effects of endogeneity, like this has been applied in HE models of chronic obstructive pulmonary disease [59].
3. When pathology and an estimation of the sequence of events are of interest: Use directed acyclic graphs [60, 61] or the network approach [62] which unravel the pathological sequence of complications to find a causal diagram to guide interdependencies.
4. When running time is not a major concern, the most straightforward method is to reduce the cycle length (to, e.g., monthly cycles). As all health statuses will then be more frequently updated, the bias introduced by ordering will be reduced [15].

The distinct methods of treatment effect integration we identified (either by the change of risk factors or dummy variables) may influence what sources of evidence can be handled. Treatment effects as indicated by risk factors, could use effect estimates from either randomized control trials or real-world evidence, and enable relatively straightforward updates of such effects. However, they risk underestimating effects that run via routes other than risk factor levels. For example, the effect of sodium-glucose cotransporter-2 inhibitors on the risk of cardiovascular complications may be underestimated when modeled based on risk factor levels alone, and trial-observed hazard ratios may provide the best fit [63]. Therefore, we recommend future HE models to adopt a hybrid approach which supplement surrogate risk factors with directly observed event rate changes [64], by incorporating both event rate changes resulting from treatment-induced risk-factor-level changes through prediction models and direct event rate changes indicated by direct evidence from observations (e.g., hazard ratios from trials). Of note, double counting should be avoided by estimating the gap between the risk-factor induced and observed event rate changes, and adjusting estimated to observed event rate changes for the trial follow-up period only. Any assumptions regarding treatment effects beyond the trial follow-up period should be clearly and transparently reported [65].

Treatment switches were modeled using a transition matrix or threshold-levels of risk factors. However, switches might also be triggered by events (e.g., CVD) regardless of past medication [66]. Therefore, future studies might consider treatment switches triggered by events or tracker variables. Furthermore, many HE models did not adequately integrate treatment compliance and persistence, which potentially affects the estimated cost-effectiveness of treatment. Future studies might incorporate compliance by establishing rates of disease progression (e.g., transition probabilities) or risk factor levels as functions of individual compliance [67].

HE models are becoming increasingly complicated and have integrated more interdependent prediction models, so transparency has become more difficult and important to achieve. Although the continuous-time model, Archimedes, was successfully validated with 18 trials (correlation = 0.99) [68], it has been criticized because of its high complexity and low transparency [37], indicating the necessity of balancing transparency and complexity when incorporating prediction models. Although there are reporting and transparency guidelines or checklists for HE models [69], prediction models [70], and diabetes modeling in particular [65], these guidelines neglect aspects valuable in estimating prediction models with the purpose of subsequently using them in HEs. These aspects include the order and interdependency of prediction models (i.e., how to order the interdependent prediction models to reflect causal relations of diabetes complications). The Diabetes Modelling Input Checklist [65] might be applied to improve model transparency. This requires clearly describing the assumption of treatment effect and the source of the risk equations for the model. Additionally, reporting the method of integrating prediction models and possible recalibration might be helpful. Furthermore, attending networks, such as the Mount Hood Diabetes Challenge Network; maintaining model registries; and reporting results from reference case simulations will improve transparency and confidence in models and ultimately improve decision-making [71].

Despite the study’s strengths, it has limitations. First, only one reviewer screened searches and selected papers. However, compared to other reviews, we did not miss any models to the best of our knowledge. Second, contrary to other reviews, we did not assess HE models’ quality but rather focused on the methodology. To get overview of all methods and prediction models applied, we did not restrict the time of publishing or the HE model’s validity. Most HE models we investigated were validated internally and externally with a satisfactory quality [7, 20, 37]. Previous studies could be consulted if the validity [7, 20, 37], quality [7, 20, 37], and suitability [20] of T2D HE models are the primary areas of interest. Finally, we could not identify an existing categorization of methods to combine prediction models into HE models and hence had to use our own terminology.

In conclusion, descriptions of prediction model integration methods in HE models tend to be ambiguous, while methods used to combine them seemed somewhat outdated, creating the need for clarification and improvement. We sought to mitigate this need by addressing the gap in assessing how prediction models that calculate complication risks are incorporated in T2D HE models. Currently, an increasing number of T2D HE models are being developed and updated for a wide range of countries, populations, complications, treatments, and indicators, enhancing the need for proper integration of prediction models. Thus, more attention should be focused on the methodology of choosing, adjusting, and ordering prediction models and the transparency of these approaches.

## Supplementary Information

Below is the link to the electronic supplementary material.

Supplementary file1 (PDF 452 kb)
