# Supplementary material for: Prediction of complications in health economic models of type 2 diabetes: a review of methods used
Source: Acta Diabetol. 2023 Mar 3;60(7):861–79. doi: 10.1007/s00592-023-02045-8 (PMC10198865; doi:10.1007/s00592-023-02045-8)
Supplement: Supplementary file 1 — Supplementary file1 (PDF 452 kb) [file 592_2023_2045_MOESM1_ESM.pdf]

Supplementary Information for

Prediction of Complications in Health Economic Models of Type 2 Diabetes: A Review of Methods Used

Acta Diabetologica

Xinyu Li, Mphil (ORCID: 0000-0002-0225-6937)[1], Fang Li, MSc (0000-0002-2651-8401)[1], Junfeng Wang, Ph.D (0000-0001-5157-5355)[2] Anoukh van Giessen, Ph.D (0000-0003-4521-9500)[3], Talitha L. Feenstra, Ph.D (0000-0002-5788-0454) [1]

[1] University of Groningen, Faculty of Science and Engineering, Groningen Research Institute of Pharmacy, Groningen, The Netherlands

[2] Division of Pharmacoepidemiology and Clinical Pharmacology, Utrecht Institute for Pharmaceutical Sciences, Utrecht University, Utrecht, The Netherlands

[3] Expertise Center for Methodology and Information Services, National Institute for Public Health and the Environment, Bilthoven, the Netherlands

Contact information for corresponding author

Xinyu Li, Mphil

Address: Groningen Research Institute of Pharmacy, A. Deusinglaan1, 9713AV, Groningen, Netherlands.

Email: li.xinyu@rug.nl

Phone: (+31) 627574221

Table of Contents

Supplementary Table 1. PRISMA-ScR Checklist[1] 3

Supplementary Table 2. Overview of type 2 diabetes decision models participating in the Mount Hood Challenges or registering with the Diabetes simulation modeling database. (14 further models were identified through published papers only) 7

Supplementary Table 3. Overview of characteristics of health economic models for type 2 diabetes mellitus 10

Supplementary Table 4. Overview of characteristics of prediction models incorporated in type 2 diabetes mellitus health economic models 19

Supplementary Appendix 1. Search Terms 27

Supplementary Appendix 2. Eastman's incidence rate, hazard ratio and transition probability algorithm[44] 29

Supplementary Appendix 3. Algorithms to convert risks from prediction models into annual rates 30

Supplementary Appendix 4. Algorithms for prediction models in EAGLE [23] 31

References 32

Supplementary Table 1. PRISMA-ScR Checklist[1]

SECTION	ITEM	PRISMA-ScR CHECKLIST ITEM	REPORTED ON PAGE
**TITLE**			
**Title**	1	Identify the report as a scoping review.	Title page
**ABSTRACT**			
**Structured summary**	2	Provide a structured summary that includes (as applicable): background, objectives, eligibility criteria, sources of evidence, charting methods, results, and conclusions that relate to the review questions and objectives.	Abstract page
**INTRODUCTION**			
**Rationale**	3	Describe the rationale for the review in the context of what is already known. Explain why the review questions/objectives lend themselves to a scoping review approach.	Introduction (Paragraphs 1-5)
**Objectives**	4	Provide an explicit statement of the questions and objectives being addressed with reference to their key elements (e.g., population or participants, concepts, and context) or other relevant key elements used to conceptualize the review questions and/or objectives.	Introduction (Paragraph 5)
**METHODS**			
**Protocol and registration**	5	Indicate whether a review protocol exists; state if and where it can be accessed (e.g., a Web address); and if available, provide registration information, including the registration number.	Methods (Paragraph 1)

**Eligibility criteria**	6	Specify characteristics of the sources of evidence used as eligibility criteria (e.g., years considered, language, and publication status), and provide a rationale.	Methods (Inclusion and Exclusion Criteria section)
**Information sources***	7	Describe all information sources in the search (e.g., databases with dates of coverage and contact with authors to identify additional sources), as well as the date the most recent search was executed.	Methods (Literature Search section)
**Search**	8	Present the full electronic search strategy for at least 1 database, including any limits used, such that it could be repeated.	Methods (Literature Search section - Appendix S1)
**Selection of sources of evidence†**	9	State the process for selecting sources of evidence (i.e., screening and eligibility) included in the scoping review.	Methods (Literature Search section)
**Data charting process‡**	10	Describe the methods of charting data from the included sources of evidence (e.g., calibrated forms or forms that have been tested by the team before their use, and whether data charting was done independently or in duplicate) and any processes for obtaining and confirming data from investigators.	Methods (Extracted Information section)
**Data items**	11	List and define all variables for which data were sought and any assumptions and simplifications made.	Methods (Extracted Information section)
**Critical appraisal of individual sources of evidence§**	12	If done, provide a rationale for conducting a critical appraisal of included sources of evidence; describe the	NA

		methods used and how this information was used in any data synthesis (if appropriate).	
**Synthesis of results**	13	Describe the methods of handling and summarizing the data that were charted.	Methods (Extracted Information section)
**RESULTS**			
**Selection of sources of evidence**	14	Give numbers of sources of evidence screened, assessed for eligibility, and included in the review, with reasons for exclusions at each stage, ideally using a flow diagram.	Results (Paragraph 1 & Fig. 1)
**Characteristics of sources of evidence**	15	For each source of evidence, present characteristics for which data were charted and provide the citations.	Results (Table 1 and Table S3)
**Critical appraisal within sources of evidence**	16	If done, present data on critical appraisal of included sources of evidence (see item 12).	NA
**Results of individual sources of evidence**	17	For each included source of evidence, present the relevant data that were charted that relate to the review questions and objectives.	Results
**Synthesis of results**	18	Summarize and/or present the charting results as they relate to the review questions and objectives.	Results (Tables 2-6 and Fig.2-4)
**DISCUSSION**			
**Summary of evidence**	19	Summarize the main results (including an overview of concepts, themes, and types of evidence available), link to the review questions and objectives, and consider the relevance to key groups.	Discussion
**Limitations**	20	Discuss the limitations of the scoping review process.	Discussion (Penultimate paragraph)

**Conclusions**	21	Provide a general interpretation of the results with respect to the review questions and objectives, as well as potential implications and/or next steps.	Discussion (Last paragraph)
**FUNDING**			
**Funding**	22	Describe sources of funding for the included sources of evidence, as well as sources of funding for the scoping review. Describe the role of the funders of the scoping review.	No funding

Supplementary Table 2. Overview of type 2 diabetes decision models participating in the Mount Hood Challenges or registering with the Diabetes simulation modeling database. (14 further models were identified through published papers only)

	1st Challenge (2000)[2]	2nd Challenge (2002)	3rd Challenge (2003)	4th Challenge (2004)[3]	5th Challenge (2010)[4]	6th Challenge (2012)	7th Challenge (2014)	8th Challenge (2016)[5]	9th Challenge (2018)[6, 7]	12th Challenge (2022)	Model registry	Current study	The reason of not being included in the current study
GDM[8]	Y	Y	Y									Y	
IMIB[9, 10]	Y											Y	
CDC[11-13]		Y		Y	Y	Y	Y		Y		Y	Y	
IMOR		Y	Y									N	No publicly accessible paper about the model found
IQVIA-CORE [14]		Y	Y	Y	Y	Y	Y	Y	Y		Y	Y	
Michigan[15]		Y	Y		Y		Y	Y	Y	Y	Y	Y	
UKPDS risk engine[16]		Y	Y	Y	Y							Y/N	Included as prediction models
Archimedes[17, 18]			Y	Y			Y				Y	Y	
Framingham risk score [19-21]			Y									Y/N	Included as prediction models
Cardiff[22]				Y	Y	Y	Y	Y	Y	Y	Y	Y	
EAGLE[23]				Y								Y	
Sheffield[24]				Y								Y	
UKPDS OM[16, 25]				Y	Y	Y	Y	Y	Y	Y	Y	Y	
ECHO[26]					Y	Y	Y	Y	Y	Y	Y	Y	
EBMI simulator[27]					Y						Y	N	No publicly accessible paper about the model found (only abstract available, and code website is no longer updated)

DMAF						Y	Y					N	No publicly accessible paper about the model found
MICADO [28, 29]						Y	Y	Y	Y	Y	Y	Y	
Reference model						Y	Y				Y	N	No publicly accessible paper about the model found
ODEM[30]							Y	Y				Y	
SPHR [31]								Y	Y		Y	Y	
TTM[32]								Y	Y	Y	Y	Y	
MMUs								Y				N	No publicly accessible paper about the model found
BRAVO[33]									Y		Y	Y	
PROSIT[34]									Y		Y	Y	
SHARP									Y			N	No publicly accessible paper about the model found
ASCEND [35, 36]										Y	Y	N	No publicly accessible paper about the model found. (Only information about cost and quality of life but not decision model is found.)
CHIME[37]										Y	Y	Y	
DOMUS[38]										Y	Y	N	No publicly accessible paper about the model found (only an abstract is found.)
IHE [39, 40]										Y	Y	Y	
PRIME[41]										Y		Y	
COSMO										Y	Y	N	Type 1 diabetes model
ECHO-T1DM										Y	Y	N	Type 1 diabetes model
Syreon[42]											Y	Y	
Number of models included	2	6	7	8	8	8	11	10	12	13	21	20	

List was made based on an overview publication [43] and the Mount Hood Challenge website (mthooddiabeteschallenge.com)

“Y” indicates “Yes” and “N” indicates “No” (The health economic model is not included in our study.)

Archimedes, Archimedes model; ASCEND, diabetes model based on A Study of Cardiovascular Events iN Diabetes; BRAVO, Building, Relating, Assessing, and Validating Outcomes diabetes microsimulation model; Cardiff, The Cardiff Diabetes Model; CDC, an economic evaluation model published by The CDC Diabetes Cost-effectiveness Group; CHIME, Chinese Hong Kong Integrated Modeling and Evaluation; COSMO, a Comprehensive Outcomes Simulation Model for patients with type 1 diabetes; DMAF, Diabetes Modelling and Analysis Framework; DOMUS, Development and Validation of the Diabetes Outcome Model for the U.S.; EAGLE, Economic Assessment of Glycemic control and Long-term Effects of diabetes model; EBMI, evidence based medicine integrator simulator; ECHO, The Economic and Health Outcomes Model of Type 2 Diabetes Mellitus; GDM, The Global Diabetes Model; IHE, The Swedish Institute for Health Economics Diabetes Cohort Model; IMIB, an economic evaluation model published by Palmer (Institute for Medical Informatics and Biostatistics) et al.; IMOR, Institute for Medical Outcomes Research; IQVIA-CORE, The IQVIA CORE Diabetes Model; MICADO, the Modelling Integrated Care for Diabetes based on Observational data; Michigan, The Michigan Model for Diabetes; ODEM, Ontario Diabetes Economic Model; PRIME, PRIME Type 2 Diabetes Model; PROSIT, the PROSIT Disease Modelling Community; SHARP Model, Study for Heart and Renal Protection Model; Sheffield, the Sheffield type 2 diabetes model; SPHR, School for Public Health Research Diabetes Model; Syreon, Syreon Diabetes Control Model; T1DM, Type 1 Diabetes Mellites; TTM, the Treatment Transitions Model; UKPDS-OM, The UK Prospective Diabetes Study Outcomes Model; UKPDS, The UK Prospective Diabetes Study risk engine; UKPDS-OM, The UK Prospective Diabetes Study Outcomes Model

Supplementary Table 3. Overview of characteristics of health economic models for type 2 diabetes mellitus

Study/model	Basic model structure (individual/cohort level, continuous/discrete time, state transition model/discrete event simulation/other)<br>Time horizon<br>Cycle length	Method of combining prediction models for each module	Macrovascular complications<br>States/Events<br>Model	Microvascular complications<br>States/Events<br>Model	Diabetes related Mortality<br><br>Other mortality, if reported
Eastman, 1997 [44, 45]	Micro-State transition (individual-level, discrete time, state transition model)<br>Lifetime<br>Annual	Simultaneously<br>No interdependency	2 CVD states<br>Multivariate model	5 Retinopathy states<br>4 Nephropathy states<br>3 Neuropathy states<br>Hazard rate from references	Multivariate model<br><br>Age-, sex-, and race-specific US mortality without diabetes - multivariate model calculated CVD risk
Brown, 2000 (GDM) [8]	Micro-Discrete event (individual-level, discrete time, discrete event simulation model)<br>(group mode embedded by user's selection)<br>1-20 years by input<br>Annual	Macrovascular (initial)<br>'Sunflower method' (post-initial)<br>Interdependent, Simultaneous evaluation (Lagged events)<br><br>Microvascular<br>Simultaneously<br>No interdependency	CHD, MI, angina, stroke, CHF, PVD<br>Initial<br>Framingham risk models<br>Post-initial<br>Hazard function	5 Retinopathy states<br>4 Nephropathy states<br>3 Neuropathy states<br>Eastman	Multivariate model<br>(The cause was defined and split CHD, CHF, stroke and non-CVD by random number and estimated risk)
Caro, 2000 [46, 47]	Micro-State transition (individual-level, discrete time, state transition model)<br>Lifetime<br>Annual	Simultaneously<br>No interdependency	MI, stroke<br>Framingham risk models	4 Retinopathy states<br>3 Nephropathy states<br>4 Neuropathy states<br>Risk functions developed from DCCT and large epidemiologic study	Age- and gender-dependent mortality adjusted for diabetes and nephropathy

Palmer, 2000<br>(IMIB) [9, 10]	Cohort-State transition<br>(cohort-level, discrete time, state transition model)<br>80 years or shorter defined by input<br>Annual	Simultaneously<br>No interdependency	8 AMI states<br>5 Stroke states<br>Framingham risk models	5 Retinopathy states<br>10 Nephropathy states<br>5 Amputation states<br>Reference for each progression	Calculated in each sub model<br><br>Mortality statistics in Switserland
Bagust, 2001<br>(DiDACT) [48]	Cohort-State transition<br>(cohort-level, discrete time, state transition model)<br>Lifetime<br>5-year band (using algebraic formulations to preserve the accuracy of annual transitions)	Simultaneously<br>No interdependency	CVD (CHD, stroke)<br>Framingham risk models	9 Retinopathy states<br>4 Nephropathy states<br>7 Neuropathy states<br>Linear function of HbA1c with option to use Eastman rate	Framingham equations<br><br>OPCS mortality statistics in UK<br><br>(Competing risk models were applied to combine mortality elements)
CDC Group, 2002<br>(CDC) [11-13]	Cohort-State transition<br>(cohort-level, discrete time, state transition model)<br>Lifetime or age 95 years<br>Annual	Simultaneously<br>No interdependency	6 CHD states<br>4 Stroke states<br>Framingham risk models (from the 2009 version of CDC model these are replaced by UKPDS 68)	3 Retinopathy states<br>5 Nephropathy states<br>6 Neuropathy states<br>Reference for each progression includes Eastman	Calculated in each sub model<br><br>(Competing risk were considered by increased mortality rates among persons with diabetes. These were attributed to increased mortality due to cardiovascular disease, ESRD, LEA, and other causes.)
David, 2003<br>(Archimedes) [17, 18]	Micro-Continuous mathematical model<br>(individual-level, continuous time, object-oriented model)	Simultaneously<br>Interdependent evaluation<br>(As in real organ systems)	Physiology-based equations		

	Flexible (up to lifetime)<br>Continuous				
Clarke, 2004 (UKPDS OM1) [16]	Micro-Discrete event (individual-level, discrete time, discrete event simulation model)<br>Lifetime<br>Annual	Random order<br>Interdependent	IHD, MI, CHF, stroke<br>UKPDS 68	Amputation, blindness, renal failure<br>UKPDS 68	UKPDS 68<br><br>UKPDS 68 estimates death from other causes (primarily cancers and accidents)
Palmer, 2004 (IQVIA-CORE) [14]	Micro-State transition (individual-level, discrete time, state transition model)<br>Lifetime or by input (1-90 years)<br>Annual (except foot ulcer 1 month and hypoglycemia 3 months)	Random order<br>Interdependent	3 CHF states<br>2 PVD states<br>Framingham (logistic)<br>2 Angina states<br>Framingham (Proportional)<br>3 stroke states<br>3 MI states<br>Framingham or UKPDS by user selection	2 Neuropathy states<br>9 Foot ulcer states<br>4 Retinopathy states<br>3 Cataract states<br>7 Nephropathy states<br>Reference for each progression	Calculated in each sub model<br><br>US mortality statistics from 1999 indexed by age and ethnic group
Zhou, 2005 (Michigan) [15]	Micro-State transition (individual-level, discrete time, state transition model)<br>Lifetime<br>Annual	Simultaneously<br>No interdependency	5 CHD states<br>4 Stroke states<br>Reference for each progression includes UKPDS 56 and 60	5 Retinopathy states<br>6 Nephropathy states<br>3 Neuropathy states<br>Reference for each progression	Calculated in each sub model<br><br>Age, sex and race specific mortality for US population<br><br>(Competing risk are considered by taking the highest risk for all fatal modules)
Mueller, 2006 (EAGLE) [23]	Micro-Discrete event (individual-level, discrete time, discrete event simulation model)<br>User defined by input<br>Annual	Simultaneously<br>No interdependency	4 Cardiovascular system events<br>(non-fatal MI, angina pectoris, HF, stroke)<br>EAGLE risk equations<br>MI and stroke with UKPDS option by user selection	6 Ophthalmic disorders events<br>3 Kidney system events<br>4 Nervous system events<br>EAGLE risk equations	EAGLE risk equations<br><br>EAGLE risk equations (with mortality rate from country specific WHO tables)

Bagust, 2006<br>(Cardiff) [22]	Micro-Discrete event (individual-level, discrete time, discrete event simulation model)<br>20 years or up to 60 years by user defined annual incidence<br>Annual	Simultaneously<br>No interdependency	CHD UKPDS56<br>Stroke UKPDS60	Retinopathy, nephropathy, neuropathy<br>Eastman	Calculated in each sub model<br><br>UK 2001-2003 gender-specific interim life tables
Grima, 2007 [49]	Cohort-State transition (cohort-level, discrete time, state transition model)<br>Lifetime or age 89<br>Annual	Simultaneously<br>No interdependency	2 Stroke states<br>2 MI states<br>2 HF states<br>Calibrate UKPDS 35 models to UKPDS 33 population	2 Renal disease states<br>2 Retinopathy states<br>2 Amputation states<br>Calibrate UKPDS 35 models to UKPDS 33 population	Calculated in model based on fatal and nonfatal proportions in UKPDS33, and HF mortality rate from a Canadian population-based study<br><br>Subtract mortality due to complications based on UKPDS 33 and 35, from all-cause mortality for the Canadian population
Tilden, 2007 [50]	Micro-State transition (individual-level, discrete time, state transition model)<br>Lifetime or age 100<br>6 months	Random order<br>Interdependent	CHF, MI, IHD, stroke<br>UKPDS68	Renal failure, blindness in one eye, amputation<br>UKPDS68	UKPDS68
Chen, 2008<br>(JADE) [51]	Micro-Discrete event (individual-level, discrete time, discrete event simulation model)<br>Lifetime or age 100<br>6 months	Random order<br>Interdependent	CHF, MI, IHD, stroke<br>UKPDS68	Renal failure, blindness, amputation<br>UKPDS68	UKPDS68
Secretariat, 2009<br>(ODEM)[30]	Micro-State transition	Random order<br>Interdependent	IHD, MI, CHF, stroke<br>UKPDS68	Amputation, blindness, renal failure<br>UKPDS68	UKPDS68

	(individual-level, discrete time, state transition model)<br>Lifetime<br>Annual				UKPDS68 estimates death from other causes (primarily cancers and accidents)
Gillett, 2010 (Sheffield) [24]	Micro-State transition (individual-level, discrete time, state transition model)<br>Lifetime<br>Annual	Simultaneously<br>No interdependency	CHD, HF, stroke<br>UKPDS56, 60, and 66	Retinopathy, nephropathy, neuropathy<br>Eastman	UKPDS68
Brändle, 2011 (DMM) [52]	Micro-Discrete event (individual-level, discrete time, discrete event simulation model)<br>40 years<br>Annual	Random order<br>Interdependent	MI, stroke, IHD<br>UKPDS68	Neuropathy (amputation), nephropathy (ESRD), retinopathy<br>UKPDS68	UKPDS68<br><br>Age-specific cause mortality minus diabetes related mortality from Swiss life tables
Hayes, 2013 (UKPDS OM2) [25]	Micro-Discrete event (individual-level, discrete time, discrete event simulation model)<br>Lifetime<br>Annual	Random order<br>Interdependent	MI, stroke, IHD, CHF<br>UKPDS82	Amputation, blindness, renal failure, ulcer<br>UKPDS82	Four separate mortality risk equations (death in years with no history or event, death in first year of event, death in years with history but no events and death in subsequent years of events)
Smolen, 2014 (TTM) [32]	Micro-Discrete event (individual-level, discrete time, discrete event simulation model)<br>5 years<br>1 month	Simultaneously<br>No interdependency	CHD, stroke<br>UKPDS56	(From CDC model)<br>Retinopathy, nephropathy, neuropathy<br>Reference for each progression includes Eastman	Calculated in each sub model<br><br>Overall natural mortality (unclear source)
IHE, 2014 [39, 40]	Cohort-State transition (cohort-level, discrete time, state transition model)	Macrovascular<br>Random order<br>Interdependent<br><br>Microvascular	100 Macrovascular health states<br>MI, IHD, CHF, stroke<br>UKPDS (68/82), NDR risk equations	120 Microvascular health states<br>Retinopathy, nephropathy, Neuropathy	UKPDS (68/82)

	40 years<br>Annual	Simultaneously<br>No interdependency		References (includes Eastman, DiDACT, GDM)	
Van Der Heijden, 2015 (MICADO) [28, 29]	Cohort-State transition (cohort-level, discrete time, state transition model)<br>Lifetime<br>Annual	Macrovascular<br>Simultaneous evaluation (lagged events)<br><br>Microvascular<br>Simultaneously<br>No interdependency	CHD, stroke, CHF, MI<br>Relative risks derived from publications	5 Retinopathy states<br>4 Nephropathy states<br>5 Foot ulcer states<br>Adapted version of Eastman	Estimated from representative national registries and systematic literature reviews<br><br>All-cause mortality from Dutch life tables (statistics Netherlands). Attributed by age and gender to different causes, while considering competing risks, using a causal diagram, systematic literature reviews for risks, and GP registries.
Nagy, 2016<br>(Syreon) [42]	Micro-State transition (individual-level, discrete time, state transition model)<br>Lifetime<br>6 months (except Hypoglycaemia, Ketoacidosis and Foot ulcer 3 months)	Unclear order, Interdependent	4 CHD states<br>3 Stroke states<br>3 PVD states<br>State transition sub model<br>Reference based transition probabilities	7 Retinopathy states<br>8 Nephropathy states<br>3 Neuropathy states<br>7 Foot ulcer states<br>State transition sub mode<br>Reference based transition probabilities	Calculated in each sub model<br><br>(Patients may die from general<br>causes, i.e., age-specific and gender-specific mortality<br>or in certain sub-models from disease-specific causes, i.e., disease-specific excess mortality.)
Schramm, 2016<br>(PROSIT) [34]	Micro-State transition (individual-level, discrete time, state transition model)<br>80 cycles<br>Annual (user definable)	Simultaneously<br>No interdependency	7 CHD states<br>MissouriB Model<br><br>5 Stroke states<br>SambesiB Model	Retinopathy<br>Jenissei B Model<br><br>5 Nephropathy states<br>ShannonB Model<br><br>Amputation<br>YukonB Model	Calculated in each sub model<br><br>Unclear
Breeze, 2017<br>(SPHR) [31]	Micro-Discrete event (individual-level, discrete time, discrete	Macrovascular<br>'Sunflower method' (independent to CHF)	CHD (angina or MI), stroke, TIA<br>Qrisk2	Foot ulcer<br>Simplified UKPDS82	Qrisk2

	event simulation model)<br>Lifetime<br>Annual	Microvascular<br>Unclear<br>Interdependent	CHF<br>Framingham risk models	Retinopathy, neuropathy<br>UKPDS82<br><br>Nephropathy<br>UKPDS68	Mortality rates by age and sex extracted from the Office for National Statistics, excluding deaths due to CVD, breast cancer, colorectal cancer and diabetes
Willis, 2017<br>(ECHO) [26]	Micro-State transition (individual-level, discrete time, state transition model)<br>User defined<br>Annual	Macrovascular<br>Random order<br>Interdependent<br>Optional 'Sunflower method' if ADVANCE or NDR was applied<br><br>Microvascular<br>Simultaneously<br>No interdependency	IHD, MI, stroke, CHF<br>User's choice UKPDS (68/82), additional option for aggregate CVD for ADVANCE and NDR-CVD risk equation	9 Retinopathy states<br>7 Neuropathy states<br>6 CKD sates<br>From references including Eastman and DiDACT	UKPDS (68/82)
Shao, 2018<br>(BRAVO) [33]	Micro-Discrete event (individual-level, discrete time, discrete event simulation model)<br>40 years<br>Annual	Random order<br>Interdependent	Stroke, CHF, MI, angina, revasc<br>BRAVO risk engine	ESRD, blindness, SPSL<br>BRAVO risk engine	BRAVO risk engine
Wu, 2018 (COMT)[53]	Micro-Discrete event (individual-level, discrete time, discrete event simulation model)<br>Lifetime<br>Annual (monthly for neuropathy)	Unclear<br>Interdependent	Stroke, MI, CHF, CVD<br>RECODe	Blindness, ESRD, clinical neuropathy, uncomplicated and complicated diabetic foot ulcer and minor and major amputation<br>RECODe	RECODe
Jiao, 2019<br>(RAMP-DM) [54]	Micro-Discrete event (individual-level, discrete time, discrete	Random order<br>Interdependent	AMI, IHD, HF, stroke<br>Derived from the observed incidences from RAMP-DM	ESRD, STDR<br>Derived from the observed incidences RAMP-DM	Derived from the observed incidences

	event simulation model)<br>Lifetime or 60 years<br>Annual				Four separate mortality risk equations (death in the years without any complications, death in the first year of events, without history, death in the first year of events, with history, and death in the years with history but not events)
Kazemian, 2019 (PREDICT-DM) [55]	Micro-State transition (individual-level, discrete time, state transition model)<br>Lifetime or 100 years<br>1 month	Unclear<br>Interdependent	MI, stroke, CHF<br>RECODe	Nephropathy<br>RECODe	Mortality from CVD (MI/stroke/CHF)<br>RECODe<br><br>Mortality from other causes (0.3*RECODe
Cornerstone Research Group, 2020 (Cornerstone) [56]	Micro-Discrete event (individual-level, discrete time, discrete event simulation model)<br>User defined up to 100 years<br>Annual	Unclear order<br>Interdependent	CHF, IHD, MI, stroke<br>UKPDS82	Blindness, ulcer, amputation, renal failure<br>UKPDS82	UKPDS82<br><br>UKPDS82
Tanaka, 2021 (JJCEM) [57]	Cohort-State transition (cohort-level, discrete time, state transition model)<br>Lifetime horizon<br>Annual	Predefined order (retinopathy – amputation)<br>Interdependent	Stroke, CHD<br>JJRE	Amputation<br>UKPDS68<br><br>Retinopathy, retinopathy progression, overt nephropathy<br>JJRE	UKPDS68<br><br>JJRE
Quan, 2021 (CHIME)[37]	Micro-Discrete event (individual-level, discrete time, discrete event simulation model)<br>Lifetime<br>Annual	Simultaneous evaluation (lagged events)<br>Interdependent	MI, IHD, HF, PVD, cerebrovascular disease<br>CHIME risk equations	Neuropathy, amputation, ulcer, renal failure, cataract, retinopathy<br>CHIME risk equations	CHIME risk equations

Pollock, 2022 (PRIME)[41]	Micro-Discrete event (individual-level, discrete time, discrete event simulation model)<br>Lifetime<br>Annual	Random order<br>Interdependent	MI, stroke, HF, IHD, Combining risk formulae using a weighted model averaging from UKPDS82, BRAVO, and Hong Kong registry risk models	Renal failure, neuropathy, amputation, foot ulcer, severe vision loss, macular edema<br>Combining risk formulae using a weighted model averaging from UKPDS82 and BRAVO	UKPDS82<br><br>UKPDS82

ADVANCE, model for cardiovascular risk prediction in Action in Diabetes and Vascular Disease: Preterax and Diamircon Modified-release Controlled Evaluation; Archimedes, Archimedes model; BRAVO, Building, Relating, Assessing, and Validating Outcomes diabetes microsimulation model; Cardiff, The Cardiff Diabetes Model; Caro, an economic evaluation model published by Caro et al.; CDC, an economic evaluation model published by The CDC Diabetes Cost-effectiveness Group; CHD, Congenital Heart Disease; CHIME, Chinese Hong Kong Integrated Modeling and Evaluation; CKD, Chronic Kidney Disease; COMT, Chinese Outcomes Model for Type 2 Diabetes; Cornerstone, Cornerstone Diabetes Simulation Model; CVD, cardiovascular disease; DCCT, Diabetes Control and Complications Trial; DiDACT, The Diabetes Decision Analysis of Cost — Type 2 model; DMM, the Diabetes Mellitus Model; DMM, the Diabetes Mellitus Model; EAGLE, Economic Assessment of Glycemic control and Long-term Effects of diabetes model; Eastman, an economic evaluation model published by Eastman et al.; ECHO, The Economic and Health Outcomes Model of Type 2 Diabetes Mellitus; ESRD, End Stage Renal Disease; GDM, The Global Diabetes Model; GP, general practitioner; Grima, an economic evaluation model published by Grima et al.; IHD, Ischemic Heart Disease; IHE, The Swedish Institute for Health Economics Diabetes Cohort Model; IMIB, an economic evaluation model published by Palmer (Institute for Medical Informatics and Biostatistics) et al.; IQVIA-CORE, The IQVIA CORE Diabetes Model; JADE, the Januvia Diabetes Economic model; JJCEM, The Japan Diabetes Complications Study/Japanese Elderly Diabetes Intervention Trial risk engine Cost-Effectiveness Model; LEA, Lower Extremity Amputation; MI, Myocardial Infarction; MICADO, the Modelling Integrated Care for Diabetes based on Observational data; Michigan, The Michigan Model for Diabetes; NDR, prediction models from Swedish National Diabetes Register; ODEM, Ontario Diabetes Economic Model; OPCS, Office of Population Censuses and Surveys; PRIME, PRIME Type 2 Diabetes Model; PROSIT, the PROSIT Disease Modelling Community; RAMP-DM, the Risk Assessment and Management Programme-Diabetes Mellitus; RECODe, Risk Equations for Complications of Type 2 Diabetes; REDICT-DM, PRojection and Evaluation of Disease Interventions, Complications, and Treatments–Diabetes Mellitus; Revasc, revascularization surgery; Sheffield, the Sheffield type 2 diabetes model; SPHR, School for Public Health Research Diabetes Model; SPSL, severe pressure sensation loss; STDR, sight-threatening diabetic retinopathy; Syreon, the Syreon Diabetes Control Model; Tilden, an economic evaluation model published by Tilden et al.; TTM, the Treatment Transitions Model; UKPDS-OM, The UK Prospective Diabetes Study Outcomes Model; UKPDS, The UK Prospective Diabetes Study risk engine; UKPDS-OM, The UK Prospective Diabetes Study Outcomes Model

Supplementary Table 4. Overview of characteristics of prediction models incorporated in type 2 diabetes mellitus health economic models

Study/model	Basic model structure	Follow up time<br>Population	Predictors	Outcomes	Treatment variable
Framingham, 1991 [19]	Parametric model (non-proportional hazards Weibull accelerated failure time model)	12 years<br><br>5,573 members of Framingham Heart Study and Framingham Offspring Study, aged 30–74 years free of CVD and cancer	Gender, age, SBP or DBP, smoking status, total cholesterol and HDL, ECG-LVH, diabetes	MI, CHD, death from CHD, stroke, CVD and death from CVD	No<br>(insulin or oral agents indicate the presence of diabetes)
Framingham-stroke, 1994 [20]	Cox proportional hazards regression model	10 years<br><br>2,372 men and 3,362 women aged 55–84 years with no history of stroke	Gender, age, SBP, dummy of antihypertensive medication, cardiovascular disease (CHD, cardiac failure, or intermittent claudication), LVH, smoking status, AF, diabetes	Stroke	Yes<br>Include the use of antihypertensive medication in the function
Framingham-CHD, 1998 [21]	Cox proportional hazards regression model	12 years<br><br>2,489 men and 2,528 women aged 30–74 years with no overt CHD	Sex, age, diabetes, smoking, blood pressure categories, and total cholesterol and LDL cholesterol categories	CHD	No<br>Treatments are not included in the formulations since it emphasizes the prediction of initial CHD events in a free-living population not on medication<br><br>(insulin or oral agents indicate the presence of diabetes)
UKPDS-35, 2000 [58]	Observational study	10 years<br><br>4,585 white, Asian Indian, and Afro-	HbA1c classifications	Any end point or deaths related to diabetes and all-cause mortality, MI, stroke, amputation (including death from	No

		Caribbean UKPDS individuals		PVD), and microvascular disease (predominantly retinal photo-coagulation), non-fatal HF and cataract extraction	
UKPDS-56, 2001 [59]	Mathematical equations based on proportional hazards models	Median 10.3 years<br><br>4,540 UKPDS DM individuals aged 25–65 years without history of MI, angina or HF	Gender, age, glycaemia, SBP and lipid levels, ethnic group, smoking status and time since diagnosis of diabetes	CHD (Fatal or non-fatal MI, or sudden death)	No<br>Not necessary because the effects of treatment were found to be consistent with effects on risk factors
UKPDS-60, 2002 [60]	Mathematical equations based on proportional hazards models fitted by maximum likelihood estimation using the Newton-Raphson method	Median 10.5 years<br><br>4,549 UKPDS DM individuals aged 25–65 years with no known stroke	Duration of diagnosed diabetes, age at diagnosis of diabetes, gender, smoking at diagnosis of diabetes, AF, SBP, lipid ratio (total cholesterol: HDL)	Stroke	No
UKPDS-66, 2004 [61]	Multivariate logistic regression model	Median 7 years<br><br>674 cases of MI occurred in 597 of 5,102 UKPDS DM individuals aged 25 to 65 years	MI (age at diagnosis of diabetes, HbA1c, SBP, time to event, urinary albumin)<br>Stroke (sex, HbA1c, SBP, subsequent stroke versus first stroke, white blood cell count)	MI or stroke being fatal	No<br>Supplementary analysis included a binary variable indicating whether individuals taking a sulfonylurea alone or in combination with other therapies showed no effect on fatal MI
UKPDS-68, 2004 [16]	Weibull proportional hazards regression	Median 10.3 years<br><br>3,642 UKPDS DM individuals aged between 25 and 65 years	Age at diagnosis of diabetes, age in years at occurrence of first diabetes-related event, time to event from diagnosis of diabetes, sex, ethnicity, smoking status, BMI, HbA1c, SBP, and lipid ratio (total	First occurrence of each of seven complications (fatal or non-fatal MI, other IHD, stroke, HF, amputation, renal failure and eye disease measured in terms of blindness in one eye) and death	No

			cholesterol:HDL), event history (including AF, PVD, IHD, MI, stroke, CHF, amputation, blindness, renal failure)		
UKPDS-82, 2013 [25]	Proportional hazards models (Exponential for MI (except first MI for female), blindness, first time amputation with prior ulcer or second time amputation and renal failure Weibull for CHF, IHD, first MI for female, stroke, first time amputation without prior ulcer Logistic regression for ulcer)	Median 17.6 years<br><br>5,102 UKPDS DM individuals aged between 25 and 65 years	Current age, duration of diabetes, sex, ethnicity, smoking status, SBP, HbA1c, LDL, HDL, BMI, eGFR, heart rate, AF, PVD, albuminuria, haemoglobin, white blood cells, event history (including MI, stroke, IHD, CHF, blindness, amputation, renal failure, diabetic ulcer)	Death, first MI, second MI, first stroke, second stroke, CHF, IHD, first amputation, second amputation, blindness, renal failure, ulcer	No
NDR-CVD, 2011 [62]	Cox proportional hazards regression model	5 years<br><br>24,288 T2DM individuals in the NDR aged 30–74 years, 15.3% with previous CVD	Age at diagnosis, sex, diabetes duration, lipid ratio (total cholesterol:HDL), HbA1c, SBP, BMI, smoking status, microalbuminuria, macroalbuminuria, AF, Previous CVD	Fatal/nonfatal CVD	No<br>Modifiable risk percent can be estimated by treatment targets
NDR, 2013 [63]	Weibull proportional hazards model	5 years<br><br>29,034 T2DM individuals in the NDR, aged 30-75 at diagnosis	Age, gender, diabetes duration, smoking, SBP, DBP, HbA1c, total-to-HDL ratio (cholesterol/HDL), LDL, history of events before diagnosis, albuminuria, and BMI	AMI, HF, stroke, NAIHD (first and second)	No

EAGLE, 2006 [23]	Regression analyses (linear, exponential, and quadratic regression formulae) (details see Appendix S5)	Three data sources, including WESDR, DCCT, and UKPDS	Age, diabetes duration, gender, HbA1c, LDL, HDL, triglycerides, macular edema, physical activity, race, SBP, smoking status	Ophthalmic disorders (non-proliferative retinopathy, proliferative retinopathy, macular edema, cataract, vitreous hemorrhage, blind in one eye), kidney system (microalbuminuria, macroalbuminuria, ESRD), nervous and vascular system (neuropathy, PAD with amputation, diabetic foot syndrome), cardiovascular system (MI, angina pectoris, HF, stroke), specific-cause mortality (MI, HF, stroke, renal disease), all-cause mortality	No<br>Treatment efficacy can be defined by the user through risk factors
Hong Kong registry risk models, 2007 [64-66]	Cox proportional hazards regression model	Median ~5.5 years<br><br>7,067 Hong Kong Chinese diabetes individuals without history of HF, and without history and clinical evidence of CHD at baseline (for stroke, 7,209 Hong Kong Chinese type 2 diabetic individuals without a history of stroke at baseline)	Age, sex, smoking status, diabetes duration, eGFR, spot urine albumin-creatinine ratio, non–high-density lipoprotein cholesterol, BMI, glycated haemoglobin，blood haemoglobin, CHD event	Stroke, hospitalization for HF, CHD	No
QRisk2, 2007 [67]	Cox proportional hazards regression model	Median 6.5 years	Age, sex, smoking status, SBP, ratio of total serum cholesterol to HDL, BMI,	CVD (including MI, CHD, stroke, and transient ischemic attack)	Yes

		1.28 million individuals, aged 35-74 years, who were free of diabetes and existing cardiovascular disease	family history of CHD in first degree relative aged less than 60, area measure of deprivation, and existing treatment with antihypertensive agent		Include the use of antihypertensive treatment in the function
ADVANCE, 2011 [68]	Cox proportional hazards regression model	4.5 years<br><br>ADVANCE cohort 11,140 high risk individuals with T2DM across 20 countries in Asia, Australasia, Europe and Canada etc., at least 55 years of age at entry	Age at diagnosis, sex, diabetes duration, pulse pressure, retinopathy, AF, HbA1c, log of urinary albumin/creatinine ratio, non-HDL cholesterol, treated hypertension	CVD	Yes<br>Include blood pressure-lowering medication in the final model as a dummy variable (use of statins and aspirin was also included in variable selection, but was dropped in the final model by stepwise approach)
JJRE, 2013 [69]	Cox proportional hazards regression model	Median 7.2 years<br><br>1,748 Japanese type 2 diabetic individuals without diabetes complications other than mild diabetic retinopathy	Sex, age, HbA1c, diabetes duration, BMI, SBP, non-HDL cholesterol, albumin-to-creatinine ratio, AF, current smoker, and leisure-time physical activity	CHD, stroke, non-cardiovascular mortality, overt nephropathy	No<br>Effects of medications on vascular complications were likely to be confounded by other clinical factors
RECODe, 2017 [70]	Cox proportional hazards regression model	Median 4.7 years<br><br>9,635 individuals from ACCORD	Age, gender, ethnicity, smoking status, SBP, CVD history, medication use (blood pressure-lowering drugs, oral diabetes drugs, and anticoagulants), HbA1c, total cholesterol, HDL, serum creatinine, urine albumin: creatinine ratio	Microvascular and cardiovascular outcomes (6 stages of nephropathy, 5 stages of retinopathy, atherosclerotic cardiovascular disease, MI, stroke, CHF, CVD mortality, and all-cause mortality)	Yes<br>Include the use of blood pressure-lowering drugs, statins, anticoagulants, oral diabetes drug

BRAVO, 2018 [33]	Weibull proportional hazards model (except Gompertz for all-cause mortality and Logistic for CVD death)	Mean 4 years or median 3.7 years<br><br>10,251 individuals from ACCORD	HbA1c, SBP, LDL, BMI, age at diagnosis, severe hypoglycemia, sex, education, smoking, race, diabetes duration, history of MI, CHF, stroke, angina, revasc, blindness, event of stroke and CHF	Stroke, CHF, MI, angina, revasc, ESRD, blindness, SPSL, all-cause mortality, CVD death	No<br>Assume treatment only impact through risk factors, and they will reach treatment target after defined years and remain constant
CHIME risk equations, 2021 [37]	Parametric proportional hazards models (exponential, log-logistic, log-normal, and Weibull)	Mean 4.1 years<br><br>98,715 individuals in Hong Kong Clinical Management System from 2006 to 2017	Age, sex, diabetes status, diabetes duration, smoking status, BMI, HbA1c, SBP, DBP, HDL, LDL, cholesterol, triglycerides, eGFR, hemoglobin, and white blood cell count; medications (insulin, non-insulin hypoglycemic agent, antihypertensives, and statins); and preexisting medical conditions (AF, MI, IHD, HF, cerebrovascular diseases, PVD, neuropathy, amputation, renal failure, hemodialysis, retinopathy, cataract, and ulcer of skin)	All-cause mortality, diabetes-related macrovascular events (MI, IHD, HF, and cerebrovascular disease), microvascular events (PVD, neuropathy, amputation, ulcer of the skin, renal failure, cataracts, and retinopathy)	Yes<br>Include the use of insulin, non-insulin hypoglycemic agent, antihypertensives, and statins in the function
Other prediction models as supplementary (they are either mentioned but not applied in HE models or the most recent versions of applied prediction models)					
Cleveland, 2008 [71]	Cox proportional hazards regression model	Median 28.6 months<br><br>33,067 T2DM individuals identified in the Cleveland clinic electronic health record who were initially	HbA1c, eGFR, BMI, SBP, DBP, HDL and LDL cholesterol, triglycerides, history of CHF and CHD, smoking status, use of concomitant medications (insulin, ACE	Risk of mortality	Yes<br>Include the use of treatment in the function

		prescribed a single oral hypoglycemic agent	inhibitor/angiotensin receptor blocker aspirin, clopidogrel, or lipid-lowering drug), new diabetes, sex, race, age, and oral medication class		
Sweden, 2008 [72]	Cox proportional hazards regression model	Median 5.64 years<br><br>11,646 individuals, aged 18–70 years, from the Swedish National Diabetes Register	HbA1c, onset age of diabetes, diabetes duration, sex, BMI, smoking, SBP, and antihypertensive and lipid-reducing drugs	5-year risk of first incident fatal/non-fatal CVD	Yes<br>Include the use of antihypertensive and lipid-reducing drugs in the function
QRisk3, 2017[73]	Cox proportional hazards regression model	Median 4.4 years<br><br>7.89 million individuals aged 25-84 years	Age, gender, ethnicity, deprivation, SBP, BMI, total cholesterol: HDL cholesterol ratio, smoking, family history of CHD in a first degree relative aged less than 60 years, type 1 diabetes, type 2 diabetes, treated hypertension, rheumatoid arthritis, AF, chronic kidney disease, a measure of SBP variability (standard deviation of repeated measures), migraine, corticosteroids, systemic lupus erythematosus, atypical antipsychotics, severe mental illness, and HIV/AIDs	CVD	Yes<br>Include the use of antihypertensive treatment in the function

ACCORD, Action to Control Cardiovascular Risk in Diabetes trial; ACE, Angiotensin-Converting-Enzyme; ADVANCE, Action in Diabetes and Vascular Disease: Preterax and Diamircon Modified-release Controlled Evaluation study; ADVANCE, model for cardiovascular risk prediction in Action in Diabetes and Vascular Disease: Preterax and Diamircon Modified-release Controlled Evaluation; AF, Atrial Fibrillation; BMI, Body Mass Index; BRAVO, the prediction models of Building, Relating, Assessing, and

Validating Outcomes diabetes microsimulation model; CHD, Coronary Heart Disease; CHF, Congestive Heart Failure; CHIME, risk prediction models in Chinese Hong Kong Integrated Modeling and Evaluation; Cleveland, a risk prediction model estimated based on Cleveland clinic electronic health record; CVD, Cardiovascular Disease; DBP, Diastolic Blood Pressure; DCCT, Diabetes Control and Complications Trial; DM, Diabetes Mellitus; EAGLE, risk prediction models in Economic Assessment of Glycemic control and Long-term Effects of diabetes model; ECG, Electrocardiographic; eGFR, estimated Glomerular Filtration Rate; ESRD, End Stage Renal Disease; Framingham, Framingham risk models; HbA1c, Hemoglobine A1c; HDL, High-Density Lipoprotein; HE, Health Economic; HF, Heart Failure; HIV/AIDs, Human Immunodeficiency Virus infection and Acquired ImmunoDeficiency syndrome; IHD, Ischemic Heart Disease; JJRE, Japanese Elderly Diabetes Intervention Trial risk engine; LDL, Low-Density Lipoprotein Cholesterol; LVH, Left Ventricular Hypertrophy; MI, Myocardial Infarction; NAIHD, NonAcute Ischemic Heart Disease; NDR, Swedish National Diabetes Register study; PAD, Peripheral Artery Disease; PVD, Peripheral Vascular Disease; Qrisk, Cardiovascular Risk Score; RECODe, Risk Equations for Complications of Type 2 Diabetes; Revasc, revascularization surgery; SBP, Systolic Blood Pressure; Sweden, risk prediction models estimated based on the Swedish National Diabetes Register; UKPDS, The UK Prospective Diabetes Study; WESDR, Wisconsin Epidemiological Study of Diabetic Retinopathy

Supplementary Appendix 1. Search Terms

**PubMed (443 found)**
("Diabetes Mellitus, Type 2"[Mesh] OR "diabetes Mellitus type 2"[Title/Abstract] OR "type 2 diabetes"[Title/Abstract] OR "diabetes type 2"[Title/Abstract] OR "diabetes mellitus type two"[Title/Abstract] OR "type two diabetes"[Title/Abstract] OR "diabetes type two"[Title/Abstract] OR T2DM[Title/Abstract] OR T2D[Title/Abstract] OR "non insulin dependant"[Title/Abstract] OR "non-insulin dependant"[Title/Abstract] OR "non insulin dependent"[Title/Abstract] OR "non-insulin dependent"[Title/Abstract] OR "noninsulin dependent"[Title/Abstract] OR NIDDM[Title/Abstract] OR "late onset diabetes"[Title/Abstract] OR "adult onset diabetes"[Title/Abstract] OR IFG[Title/Abstract] OR "impaired fasting glucose"[Title/Abstract] OR IGT[Title/Abstract] OR "impaired glucose tolerance"[Title/Abstract] OR "impaired fasting glycaemia"[Title/Abstract])
AND
("Cost-Benefit Analysis"[Mesh] OR "Models, Economic"[Mesh] OR "economic model"[Title/Abstract] OR "decision model"[Title/Abstract] OR "cost-effectiveness"[Title/Abstract] OR "economic evaluation"[Title/Abstract] OR "health economic"[Title/Abstract] OR "cost effective"[Title/Abstract] OR "health-allocation"[Title/Abstract] OR "health-utilization"[Title/Abstract] OR "cost-utility"[Title/Abstract] OR "cost-benefit"[Title/Abstract] OR "cost analysis"[Title/Abstract] OR "economic impact"[Title/Abstract] OR "monetary"[Title/Abstract] OR "reimbursement"[Title/Abstract] OR "insurance"[Title/Abstract] OR (("cost"[Title/Abstract] OR "expenditure"[Title/Abstract] OR "value"[Title/Abstract]) AND ("benefit"[Title/Abstract] OR "life years"[Title/Abstract] OR "quality-adjusted life years"[Title/Abstract] OR "disability adjusted life years"[Title/Abstract] OR "utilities"[Title/Abstract] OR "utility"[Title/Abstract] "utility")))
AND
("Decision Support Techniques"[Mesh] OR "risk engine"[Title/Abstract] OR "risk score"[Title/Abstract] OR "risk equation"[Title/Abstract] OR "risk assessment"[Title/Abstract] OR "algorithm"[Title/Abstract] OR "prediction model"[Title/Abstract] OR "predictive model"[Title/Abstract] OR "predicting"[Title/Abstract] OR "prediction rule"[Title/Abstract] OR "prediction"[Title/Abstract] OR "prediction functions"[Title/Abstract])
**Embase (790 found)**
('non insulin dependent diabetes mellitus'/de OR 'diabetes mellitus type 2':ti,ab,kw OR 'type 2 diabetes':ti,ab,kw OR 'diabetes type 2':ti,ab,kw OR 'diabetes mellitus type two':ti,ab,kw OR 'type two diabetes':ti,ab,kw OR 'diabetes type two':ti,ab,kw OR t2dm:ti,ab,kw OR t2d:ti,ab,kw OR 'non insulin dependant':ti,ab,kw OR 'non-insulin dependant':ti,ab,kw OR 'noninsulin dependant':ti,ab,kw OR 'non insulin dependent':ti,ab,kw OR 'non-insulin dependent':ti,ab,kw OR 'noninsulin dependent':ti,ab,kw OR niddm:ti,ab,kw OR 'late onset diabetes':ti,ab,kw OR 'adult onset diabetes':ti,ab,kw OR ifg:ti,ab,kw OR 'impaired fasting glucose':ti,ab,kw OR igt:ti,ab,kw OR 'impaired glucose tolerance':ti,ab,kw OR 'impaired fasting glycaemia':ti,ab,kw)
AND
('economic model'/de OR 'economic model':ti,ab,kw OR 'decision model':ti,ab,kw OR 'cost-effectiveness':ti,ab,kw OR 'economic evaluation':ti,ab,kw OR 'health economic':ti,ab,kw OR 'cost effective':ti,ab,kw OR 'health-allocation':ti,ab,kw OR 'health-utilization':ti,ab,kw OR 'cost-utility':ti,ab,kw OR 'cost-benefit':ti,ab,kw OR 'cost analysis':ti,ab,kw OR 'economic impact':ti,ab,kw OR 'monetary':ti,ab,kw OR 'reimbursement':ti,ab,kw OR 'insurance':ti,ab,kw OR (('cost':ti,ab,kw OR 'expenditure':ti,ab,kw OR 'value':ti,ab,kw) AND ('benefit':ti,ab,kw OR 'life years':ti,ab,kw OR 'quality-adjusted life years':ti,ab,kw OR 'disability adjusted life years':ti,ab,kw OR 'utilities':ti,ab,kw OR 'utility':ti,ab,kw)))
AND
('risk model'/de OR 'risk engine':ti,ab,kw OR 'risk score':ti,ab,kw OR 'risk equation':ti,ab,kw OR 'risk assessment':ti,ab,kw OR 'algorithm':ti,ab,kw OR 'prediction model':ti,ab,kw OR 'predictive model':ti,ab,kw OR 'predicting':ti,ab,kw OR 'prediction rule':ti,ab,kw OR 'prediction':ti,ab,kw OR 'prediction functions':ti,ab,kw)
**Cochrane Library (195 found)**
#1 MeSH descriptor: [Diabetes Mellitus, Type 2] explode all trees
#2 MeSH descriptor: [Models, Economic] explode all trees
#3 MeSH descriptor: [Cost-Benefit Analysis] explode all trees

#4 MeSH descriptor: [Decision Support Techniques] explode all trees
#5 ("diabetes Mellitus type 2" or "type 2 diabetes" or "diabetes type 2" or "diabetes mellitus type two" or "type two diabetes" or "diabetes type two" or T2DM or T2D or "non insulin dependant" or "non-insulin dependant" or "noninsulin dependant" or "non insulin dependent" or "non-insulin dependent" or "noninsulin dependent" or NIDDM or "late onset diabetes" or "adult onset diabetes" or IFG or "impaired fasting glucose" or IGT or "impaired glucose tolerance" or "impaired fasting glycaemia"):ti,ab,kw
#6 ("risk engine" or "risk score" or "risk equation" or "risk assessment" OR "algorithm" OR "prediction model" OR "predictive model" OR "predicting" OR "prediction rule" OR "prediction" OR "prediction functions"):ti,ab,kw
#7 ("economic model" or "decision model" or "cost-effectiveness" or "economic evaluation" or “health economic” or “cost effective” OR “health-allocation” OR “health-utilization” OR “cost-utility” OR “cost-benefit” OR “cost analysis” OR “economic impact” OR “monetary” OR “reimbursement” OR “insurance” OR ((“cost” OR “expenditure” OR “value”) AND (“benefit” OR “life years” OR “quality-adjusted life years” OR “disability adjusted life years” OR “utilities” OR “utility”))):ti,ab,kw
(#1 or #5) AND (#2 or #3 or #7) AND (#4 or #6)
**Web of Science (495 found)**
TS=("diabetes Mellitus type 2" or "type 2 diabetes" or "diabetes type 2" or "diabetes mellitus type two" or "type two diabetes" or "diabetes type two" or T2DM or T2D or "non insulin dependant" or "non-insulin dependant" or "noninsulin dependant" or "non insulin dependent" or "non-insulin dependent" or "noninsulin dependent" or NIDDM or "late onset diabetes" or "adult onset diabetes" or IFG or "impaired fasting glucose" or IGT or "impaired glucose tolerance" or "impaired fasting glycaemia")
AND
TS=("economic model" or "decision model" or "cost-effectiveness" or "economic evaluation" or “health economic” or “cost effective” OR “health-allocation” OR “health-utilization” OR “cost-utility” OR “cost-benefit” OR “cost analysis” OR “economic impact” OR “monetary” OR “reimbursement” OR “insurance” OR ((“cost” OR “expenditure” OR “value”) AND (“benefit” OR “life years” OR “quality-adjusted life years” OR “disability adjusted life years” OR “utilities” OR “utility”)))
AND
TS= ("risk engine" or "risk score" or "risk equation" or "risk assessment" OR "algorithm" OR "prediction model" OR "predictive model" OR "predicting" OR "prediction rule" OR "prediction" OR "prediction functions")

Supplementary Appendix 2. Eastman's incidence rate, hazard ratio and transition probability algorithm[44]

$$Incidence\ rate\ (hazard\ rate) = \frac{log(\frac{1}{1 - The\ proportion\ of\ events\ in\ the\ population\ at\ risk})}{The\ period\ of\ observation}$$

$$The\ risk\ of\ occurence\ = 1 - e^{-hazard\ ratio \times t}$$

Supplementary Appendix 3. Algorithms to convert risks from prediction models into annual rates

1. RAMP-DM's algorithm[54] to convert 5-year cumulative incidences to an average annual rate

$$rate = -\frac{log(1 - 5\ year\ cumulative\ incidences)}{5}$$

$$Average\ annual\ transition\ probability = 1 - e^{-rate}$$

2. GDM's algorithm[8] to convert 4-year cumulative incidences to an average annual rate

$$\mu = \beta_0 + \beta_1 x_1 + \beta_2 x_2 + \cdots \beta_k x_k$$
$$log\sigma = \Theta_0 + \Theta_1 \times \mu$$

where x denotes risk factors for an individual, and β denotes coefficients estimated in the Framingham risk equations [19]

$$rate = \frac{\frac{4^{\frac{1}{\sigma}}}{e^{\mu}}}{4}$$

3. SPHR's algorithm[31] for the probability of an event in the next period
The equation for the probability of an event in the next period is calculated as

$$p(Y = 1) = 1 - S(1)^{\theta}$$

Underlying survival curves for men and women were extracted from the QRISK2 open source file, and θ is the sum product of the coefficients reported in the QRISK2 risk equation multiplied by the individual's characteristics.

Supplementary Appendix 4. Algorithms for prediction models in EAGLE [23]

The risk equations for each event were developed based on the base risk and relative risk:

$$\mathrm{P}_t(e) = \sum_{t=1}^{n} \left[\mathrm{P}_b(e)\left(\prod_{j=1}^{m} Relative\ Risk\ (x_j)\right) \right]_t$$

$\mathrm{P}_t(e)$ indicates the cumulative probability of getting and event e during the time horizon t=1 to n.

$\mathrm{P}_b(e)$ indicates the base risk for the event e.

$Relative\ Risk\ (x_j)$ indicates the relative risk associated with the specific risk parameter x_j, and it can be a linear, quadratic, or exponential function.

References

[1] Tricco AC, Zarin LE, O'Brien KKE, Colquhoun H, Levac D (2018) Preferred Reporting Items for Systematic reviews and Meta-Analyses extension for Scoping Reviews (PRISMA-ScR) Checklist. Ann Intern Med 169(7): 11-12. 10.7326/M18-0850.2

[2] Brown JB, Palmer AJ, Bisgaard P, Chan W, Pedula K, Russell A (2000) The Mt. Hood challenge: cross-testing two diabetes simulation models. Diabetes Research and Clinical Practice 50: S57-S64. Doi 10.1016/S0168-8227(00)00217-5

[3] Palmer AJ, Roze S, Valentine WJ, et al. (2007) Computer modeling of diabetes and its complications: A report on the Fourth Mount Hood Challenge Meeting. Diabetes Care 30(6): 1638-1646. 10.2337/dc07-9919

[4] Palmer AJ (2013) Computer modeling of diabetes and its complications: A report on the fifth Mount Hood challenge meeting. Value in Health 16(4): 670-685. 10.1016/j.jval.2013.01.002

[5] Palmer AJ, Si L, Tew M, et al. (2018) Computer Modeling of Diabetes and Its Transparency: A Report on the Eighth Mount Hood Challenge. Value in Health 21(6): 724-731. 10.1016/j.jval.2018.02.002

[6] Si L, Willis MS, Asseburg C, et al. (2020) Evaluating the Ability of Economic Models of Diabetes to Simulate New Cardiovascular Outcomes Trials: A Report on the Ninth Mount Hood Diabetes Challenge. Value in Health 23(9): 1163-1170. 10.1016/j.jval.2020.04.1832

[7] Tew M, Willis M, Asseburg C, et al. (2022) Exploring Structural Uncertainty and Impact of Health State Utility Values on Lifetime Outcomes in Diabetes Economic Simulation Models: Findings from the Ninth Mount Hood Diabetes Quality-of-Life Challenge. Medical Decision Making 42(5): 599-611. Artn 0272989x211065479
10.1177/0272989x211065479

[8] Brown JB, Russell A, Chan W, Pedula K, Aickin M (2000) The global diabetes model: User friendly version 3.0. Diabetes Research and Clinical Practice 50(SUPPL. 3). 10.1016/S0168-8227(00)00215-1

[9] Palmer AJ, Brandt A, Gozzoli V, Weiss C, Stock H, Wenzel H (2000) Outline of a diabetes disease management model: Principles and applications. Diabetes Research and Clinical Practice 50(SUPPL. 3): 47-56. 10.1016/S0168-8227(00)00216-3

[10] Palmer AJ, Weiss C, Sendi PP, et al. (2000) The cost-effectiveness of different management strategies for Type I diabetes: a Swiss perspective. In:

[11] The CDCDC-ESG (1998) The Cost-effectiveness of Screening for Type 2 Diabetes.

[12] Group CDCDC-E (2002) Cost-effectiveness of Intensive Glycemic and Serum Cholesterol Level Reduction for Type 2 Diabetes. Jama 287(19): 2542-2551

[13] Press R (2009) Validation of the CDC-RTI Diabetes Cost-Effectiveness Model. Published online

[14] Palmer AJ, Roze S, Valentine WJ, et al. (2004) The CORE Diabetes Model: Projecting long-term clinical outcomes, costs and cost-effectiveness of interventions in diabetes mellitus (types 1 and 2) to support clinical and reimbursement decision-making. Curr Med Res Opin 20 Suppl 1: S5-26. 10.1185/030079904X1980

[15] Zhou H, Isaman DJM, Messinger S, et al. (2005) A computer simulation model of diabetes progression, quality of life, and cost. Diabetes care 28(12): 2856-2863. 10.2337/diacare.28.12.2856

[16] Clarke PM, Gray AM, Briggs A, et al. (2004) A model to estimate the lifetime health outcomes of patients with Type 2 diabetes: The United Kingdom Prospective Diabetes Study (UKPDS) Outcomes Model (UKPDS no. 68). Diabetologia. 10.1007/s00125-004-1527-z

[17] Schlessinger L, Eddy DM (2002) Archimedes: A new model for simulating health care systems - The mathematical formulation. Journal of Biomedical Informatics 35(1): 37-50. 10.1016/S1532-0464(02)00006-0

[18] Eddy DM, Schlessinger L (2003) Archimedes - A trial-validated model of diabetes. Diabetes Care 26(11): 3093-3101. DOI 10.2337/diacare.26.11.3093
[19] Anderson KM, Odell PM, Wilson PWF, Kannel WB (1991) Cardiovascular disease risk profiles. American Heart Journal. 10.1016/0002-8703(91)90861-B
[20] D'Agostino RB, Wolf PA, Belanger AJ, Kannel WB (1994) Stroke risk profile: Adjustment for antihypertensive medication the framingham study. Stroke. 10.1161/01.STR.25.1.40
[21] Wilson PWF, D'Agostino RB, Levy D, Belanger AM, Silbershatz H, Kannel WB (1998) Prediction of coronary heart disease using risk factor categories. Circulation. 10.1161/01.CIR.97.18.1837
[22] McEwan P, Peters JR, Bergenheim K, Currie CJ (2006) Evaluation of the costs and outcomes from changes in risk factors in type 2 diabetes using the Cardiff stochastic simulation cost-utility model (DiabForecaster). Current Medical Research and Opinion 22(1): 121-129. 10.1185/030079906X80350
[23] Mueller E, Maxion-Bergemann S, Gultyaev D, et al. (2006) Development and Validation of the Economic Assessment of Glycemic Control and Long-Term Effects of Diabetes (EAGLE) Model. In. Vol 8
[24] Gillett M, Dallosso HM, Dixon S, et al. (2010) Delivering the diabetes education and self management for ongoing and newly diagnosed (DESMOND) programme for people with newly diagnosed type 2 diabetes: Cost effectiveness analysis. BMJ (Online) 341(7770). 10.1136/bmj.c4093
[25] Hayes AJ, Leal J, Gray AM, Holman RR, Clarke PM (2013) UKPDS Outcomes Model 2: A new version of a model to simulate lifetime health outcomes of patients with type 2 diabetes mellitus using data from the 30 year united kingdom prospective diabetes Study: UKPDS 82. Diabetologia 56(9): 1925-1933. 10.1007/s00125-013-2940-y
[26] Willis M, Johansen P, Nilsson A, Asseburg C (2017) Validation of the Economic and Health Outcomes Model of Type 2 Diabetes Mellitus (ECHO-T2DM). PharmacoEconomics 35(3): 375-396. 10.1007/s40273-016-0471-3
[27] Blum S, Vardi M, Brown JB, et al. (2010) Vitamin E reduces cardiovascular disease in individuals with diabetes mellitus and the haptoglobin 2-2 genotype. Pharmacogenomics 11(5): 675-684. 10.2217/Pgs.10.17
[28] van der Heijden AA, Feenstra TL, Hoogenveen RT, et al. (2015) Policy evaluation in diabetes prevention and treatment using a population-based macro simulation model: the MICADO model. Diabet Med 32(12): 1580-1587. 10.1111/dme.12811
[29] Baan CA, Bos G, Jacobs-van der Bruggen MAM (2005) Modeling chronic diseases: the diabetes module Justification of (new) input data. 1-33
[30] Medical Advisory S (2009) Application of the Ontario Diabetes Economic Model (ODEM) to Determine the Cost-effectiveness and Budget Impact of Selected Type 2 Diabetes Interventions in Ontario. Ont Health Technol Assess Ser 9(25): 1-21
[31] Breeze PR, Thomas C, Squires H, et al. (2017) The impact of Type 2 diabetes prevention programmes based on risk-identification and lifestyle intervention intensity strategies: a cost-effectiveness analysis. Diabetic medicine : a journal of the British Diabetic Association 34(5): 632-640. 10.1111/dme.13314
[32] Smolen HJ, Murphy DR, Gahn JC, Yu X, Curtis BH (2014) The Evaluation of Clinical and Cost Outcomes Associated with Earlier Initiation of Insulin in Patients with Type 2 Diabetes Mellitus. In. Vol 20
[33] Shao H, Fonseca V, Stoecker C, Liu S, Shi L (2018) Novel Risk Engine for Diabetes Progression and Mortality in USA: Building, Relating, Assaessing, and Validating Outcomes (BRAVO). PharmacoEconomics 36(9): 1125-1134. 10.1007/s40273-018-0662-1

[34] Schramm W, Sailer F, Pobiruchin M, Weiss C open source disease models for diabetes mellitus. Available from https://www.prosit.de/index.php/Main_Page
[35] Keng MJ, Leal J, Bowman L, Armitage J, Mihaylova B, Group ASC (2022) Decrements in health-related quality of life associated with adverse events in people with diabetes. Diabetes Obes Metab 24(3): 530-538. 10.1111/dom.14610
[36] Keng MJ, Leal J, Bowman L, Armitage J, Mihaylova B, Group ASC (2022) Hospital costs associated with adverse events in people with diabetes in the UK. Diabetes Obes Metab 24(11): 2108-2117. 10.1111/dom.14796
[37] Quan J, Ng CS, Kwok HHY, et al. (2021) Development and validation of the CHIME simulation model to assess lifetime health outcomes of prediabetes and type 2 diabetes in Chinese populations: A modeling study. PLoS Med 18(6): e1003692. 10.1371/journal.pmed.1003692
[38] WINN AN, HUANG E, CLARKE P, et al. (2022) 886-P: Development and Validation of the Diabetes Outcome Model for the U.S. (DOMUS). Diabetes 71(Supplement_1). 10.2337/db22-886-P
[39] Lundqvist A, Carlsson KS, Johansen P, Andersson E, Willis M (2014) Validation of the IHE cohort model of type 2 diabetes and the impact of choice of macrovascular risk equations. PLoS ONE 9(10). 10.1371/journal.pone.0110235
[40] Steen Carlsson K, Persson U (2014) Cost-effectiveness of add-on treatments to metformin in a Swedish setting: Liraglutide vs sulphonylurea or sitagplitin. Journal of Medical Economics 17(9): 658-669. 10.3111/13696998.2014.933110
[41] Pollock RF, Norrbacka K, Boye KS, Osumili B, Valentine WJ (2022) The PRIME Type 2 Diabetes Model: a novel, patient-level model for estimating long-term clinical and cost outcomes in patients with type 2 diabetes mellitus. Journal of Medical Economics 25(1): 393-402
[42] Nagy B, Zsólyom A (2016) Cost-effectiveness of a risk-based secondary screening programme of type 2 diabetes.(May): 710-729. 10.1002/dmrr
[43] Dadwani RS, Laiteerapong N (2020) Economic Simulation Modeling in Type 2 Diabetes. Current Diabetes Reports 20(7). ARTN 24
10.1007/s11892-020-01306-y
[44] Eastman RC, Javitt JC, Herman WH, et al. (1997) Model of complications of NIDDM: I. Model construction and assumptions. Diabetes Care 20(5): 725-734. 10.2337/diacare.20.5.725
[45] Eastman RC, Javitt JC, Herman WH, et al. (1997) Model of Complications of NIDDM II. Analysis of the health benefits and cost-effectiveness of treating NIDDM with the goal of normoglycemia. 20(5)
[46] Caro JJ, Klittich WS, Raggio G, et al. (2000) Economic assessment of troglitazone as an adjunct to sulfonylurea therapy in the treatment of type 2 diabetes. Clinical Therapeutics 22(1): 116-127. 10.1016/S0149-2918(00)87983-7
[47] Caro JJ, Ward AJ, O'Brien JA (2002) Lifetime costs of complications resulting from type 2 diabetes in the US. Diabetes Care 25(3): 476-481. DOI 10.2337/diacare.25.3.476
[48] Bagust A, Hopkinson PK, Maier W, Currie CJ (2001) An economic model of the long-term health care burden of Type II diabetes. Diabetologia 44(12): 2140-2155. 10.1007/s001250100023
[49] Grima DT, Thompson MF, Sauriol L (2007) Modelling cost effectiveness of insulin glargine for the treatment of type 1 and 2 diabetes in Canada. PharmacoEconomics 25(3): 253-266. 10.2165/00019053-200725030-00007
[50] Tilden DP, Mariz S, O'Bryan-Tear G, Bottomley J, Diamantopoulos A (2007) A Lifetime Modelled Economic Evaluation Comparing Pioglitazone and Rosiglitazone for the Treatment of Type 2 Diabetes Mellitus in the UK. In. Vol 25, pp 39-54

[51] Chen J, Alemao E, Yin D, Cook J (2008) Development of a diabetes treatment simulation model: With application to assessing alternative treatment intensification strategies on survival and diabetes-related complications. Diabetes, Obesity and Metabolism 10(SUPPL.1): 33-42. 10.1111/j.1463-1326.2008.00885.x
[52] Brändle M, Azoulay M, Greiner RA (2011) Cost-effectiveness of insulin glargine versus NPH insulin for the treatment of Type 2 diabetes mellitus, modeling the interaction between hypoglycemia and glycemic control in Switzerland. International Journal of Clinical Pharmacology and Therapeutics 49(3): 217-230. 10.5414/CPP49217
[53] Wu B, Ma J, Zhang S, Zhou L, Wu H (2018) Development and validation of a Health Policy Model of Type 2 diabetes in Chinese setting. Journal of Comparative Effectiveness Research 7(8): 749-763. 10.2217/cer-2018-0001
[54] Jiao F, Wan EYF, Fung CSC, et al. (2019) Cost-effectiveness of a primary care multidisciplinary Risk Assessment and Management Program for patients with diabetes mellitus (RAMP-DM) over lifetime. Endocrine 63(2): 259-269. 10.1007/s12020-018-1727-9
[55] Kazemian P, Wexler DJ, Fields NF, Parker RA, Zheng A, Walensky RP (2019) Development and Validation of PREDICT-DM: A New Microsimulation Model to Project and Evaluate Complications and Treatments of Type 2 Diabetes Mellitus. Diabetes Technology & Therapeutics 21(6): 344-355. 10.1089/dia.2018.0393
[56] Su ZT, Bartelt-Hofer J, Brown S, et al. (2020) The Use of Computer Simulation Modeling to Estimate Complications in Patients with Type 2 Diabetes Mellitus: Comparative Validation of the Cornerstone Diabetes Simulation Model. PharmacoEconomics - open 4(1): 37-44. 10.1007/s41669-019-0156-x
[57] Tanaka S, Langer J, Morton T, et al. (2021) Developing a health economic model for Asians with type 2 diabetes based on the Japan Diabetes Complications Study and the Japanese Elderly Diabetes Intervention Trial. BMJ OPEN DIABETES RESEARCH & CARE 9(1). 10.1136/bmjdrc-2021-002177
[58] Stratton IM, Adler AI, Neil HAW, et al. (2000) Association of glycaemia with macrovascular and microvascular complications of type 2 diabetes (UKPDS 35): prospective observational study. Bmj-British Medical Journal 321(7258): 405-412. DOI 10.1136/bmj.321.7258.405
[59] Stevens RJ, Kothari V, Adler AI, Stratton IM, Holman RR (2001) The UKPDS risk engine: A model for the risk of coronary heart disease in type II diabetes (UKPDS 56). Clinical Science. 10.1042/CS20000335
[60] Kothari V, Stevens RJ, Adler AI, et al. (2002) UKPDS 60: Risk of stroke in type 2 diabetes estimated by the UK Prospective Diabetes Study risk engine. Stroke. 10.1161/01.STR.0000020091.07144.C7
[61] Stevens RJ, Coleman RL, Adler AI, Stratton IM, Matthews DR, Holman RR (2004) Risk Factors for Myocardial Infarction Case Fatality and Stroke Case Fatality in Type 2 Diabetes: UKPDS 66. Diabetes Care. 10.2337/diacare.27.1.201
[62] Zethelius B, Eliasson B, Eeg-Olofsson K, Svensson AM, Gudbjörnsdottir S, Cederholm J (2011) A new model for 5-year risk of cardiovascular disease in type 2 diabetes, from the Swedish National Diabetes Register (NDR). Diabetes Research and Clinical Practice. 10.1016/j.diabres.2011.05.037
[63] Kiadaliri AA, Gerdtham UG, Nilsson P, Eliasson B, Gudbjornsdottir S, Carlsson KS (2013) Towards Renewed Health Economic Simulation of Type 2 Diabetes: Risk Equations for First and Second Cardiovascular Events from Swedish Register Data. Plos One 8(5). ARTN e62650
10.1371/journal.pone.0062650

[64] Yang X, So WY, Kong APS, et al. (2007) Development and validation of stroke risk equation for Hong Kong Chinese patients with type 2 diabetes - The Hong Kong Diabetes Registry. Diabetes Care 30(1): 65-70. 10.2337/dc06-1273
[65] Yang XL, Ma RC, So WY, et al. (2008) Development and validation of a risk score for hospitalization for heart failure in patients with Type 2 Diabetes Mellitus. Cardiovascular Diabetology 7. Artn 9
10.1186/1475-2840-7-9
[66] Yang X, So WY, Kong APS, et al. (2008) Development and validation of a total coronary heart disease risk score in type 2 diabetes mellitus. American Journal of Cardiology 101(5): 596-601. 10.1016/j.amjcard.2007.10.019
[67] Hippisley-Cox J, Coupland C, Vinogradova Y, Robson J, May M, Brindle P (2007) Derivation and validation of QRISK, a new cardiovascular disease risk score for the United Kingdom: Prospective open cohort study. British Medical Journal. 10.1136/bmj.39261.471806.55
[68] Kengne AP, Patel A, Marre M, et al. (2011) Contemporary model for cardiovascular risk prediction in people with type 2 diabetes. European Journal of Cardiovascular Prevention and Rehabilitation. 10.1177/1741826710394270
[69] Tanaka S, Tanaka S, Iimuro S, et al. (2013) Predicting macro- and microvascular complications in type 2 diabetes: The japan diabetes complications study/the japanese elderly diabetes intervention trial risk engine. Diabetes Care 36(5): 1193-1199. 10.2337/dc12-0958
[70] Basu S, Sussman JB, Berkowitz SA, Hayward RA, Yudkin JS (2017) Development and validation of Risk Equations for Complications Of type 2 Diabetes (RECODe) using individual participant data from randomised trials. Lancet Diabetes & Endocrinology 5(10): 788-798. 10.1016/S2213-8587(17)30221-8
[71] Wells BJ, Jain A, Arrigain S, Yu C, Rosenkrans WA, Rattan MW (2008) Predicting 6-year mortality risk in patients with type 2 diabetes. Diabetes Care. 10.2337/dc08-1047
[72] Cederholm J, Eeg-Olofsson K, Eliasson B, Zethelius B, Nilsson PM, Gudbjörnsdottir S (2008) Risk prediction of cardiovascular disease in type 2 diabetes: a risk equation from the Swedish National Diabetes Register. Diabetes care
[73] Hippisley-Cox J, Coupland C, Brindle P (2017) Development and validation of QRISK3 risk prediction algorithms to estimate future risk of cardiovascular disease: prospective cohort study. BMJ 357: j2099. 10.1136/bmj.j2099
[74] McEwan P, Gordon J, Evans M, Ward T, Bennett H, Bergenheim K (2015) Estimating Cost-Effectiveness in Type 2 Diabetes: The Impact of Treatment Guidelines and Therapy Duration. Medical decision making : an international journal of the Society for Medical Decision Making 35(5): 660-670. 10.1177/0272989X14565821
[75] McEwan P, Bennett H, Khunti K, et al. (2020) Assessing the cost-effectiveness of sodium-glucose cotransporter-2 inhibitors in type 2 diabetes mellitus: A comprehensive economic evaluation using clinical trial and real-world evidence. Diabetes Obesity & Metabolism 22(12): 2364-2374. 10.1111/dom.14162
[76] Association AD (2021) 9. Pharmacologic approaches to glycemic treatment: Standards of Medical Care in Diabetes—2021. Diabetes Care 44(Supplement 1): S111-S124
[77] Hughes D, Cowell W, Koncz T, Cramer J (2007) Methods for integrating medication compliance and persistence in pharmacoeconomic evaluations. In. Vol 10. Elsevier Ltd, pp 498-509
